# Diazocine-functionalized TATA platforms

**DOI:** 10.3762/bjoc.15.150

**Published:** 2019-07-05

**Authors:** Roland Löw, Talina Rusch, Fynn Röhricht, Olaf Magnussen, Rainer Herges

**Affiliations:** 1 Otto Diels Institute of Organic Chemistry, University of Kiel, Otto-Hahn-Platz 4, 24118 Kiel, Germany; 2 Institute for Experimental and Applied Physics, University of Kiel, Leibnizstraße 19, 24098 Kiel, Germany

**Keywords:** *cis*–*trans* isomerization, diazocine, molecular switch, photochrome, self-assembled monolayers, TATA platform

## Abstract

Recently, it has been shown that the thermochemical *cis*→*trans* isomerization of azobenzenes is accelerated by a factor of more than 1000 by electronic coupling to a gold surface via a conjugated system with 11 bonds and a distance of 14 Å. The corresponding molecular architecture consists of a platform (triazatriangulenium (TATA)) which adsorbs on the gold surface, with an acetylene spacer standing upright, like a post in the middle of the platform and the azobenzene unit mounted on top. The rate acceleration is due to a very peculiar thermal singlet–triplet–singlet mechanism mediated by bulk gold. To investigate this mechanism further and to examine scope and limitation of the “spin-switch catalysis” we now prepared analogous diazocine systems. Diazocines, in contrast to azobenzenes, are stable in the *cis*-configuration. Upon irradiation with light of 405 nm the *cis*-configuration isomerizes to the *trans*-form, which slowly returns back to the stable *cis*-isomer. To investigate the thermal *trans*→*cis* isomerization as a function of the conjugation to the metal surface, we connected the acetylene spacer in *meta* (weak conjugation) and in *para* (strong conjugation) position. Both isomers form ordered monolayers on Au(111) surfaces.

## Introduction

Catalysts increase chemical reaction rates by lowering the activation energies and thus create more favorable reaction pathways [1–4]. However, there are very few reactions which do not follow the classical Eyring theory [5–6]. The rate of these reactions is not dependent on an activation barrier but controlled by quantum mechanical transition probabilities between two quantum states [7–10]. The majority of these quantum chemically forbidden reactions are photochemical processes or transition metal reactions including transitions between spin states or electronic states. We recently discovered a purely organic system in the ground state, whose reaction rate is accelerated from days to seconds by electronic coupling to a bulk gold surface via a conjugated linker over 11 bonds and 14 Å [11]. Thermal *cis*→*trans* isomerizations of azobenzenes are usually slow with half-lives of the *trans*-isomer within the range of hours to days at room temperature (parent azobenzene: 4–5 d at 25 °C) [12]. Rotation around the N=N bond is a symmetry-forbidden process and the slow isomerization proceeds via inversion at the N atoms [13]. The rate of isomerization is temperature dependent and follows a classical Arrhenius type behavior [12]. However, the rate and the mechanism change dramatically if the azobenzenes are electronically coupled to bulk gold [14–17]. To investigate the *cis*→*trans* isomerizations of azobenzenes as a function of electronic coupling systematically, we used the so-called platform approach [18]. The azobenzenes are not directly adsorbed on the surface, but covalently mounted on “TATA” (triazatriangulenium) platforms which adsorb on Au(111) surfaces. A spacer, such as an ethynyl group is connected to the central carbon atom like a post and the azobenzene is mounted on top of the spacer. After preparation of an ordered self-assembled monolayer on gold, the azobenzene units are freestanding upright on the surface. The platform defines the lateral distance between next neighbors and provides the free volume for unhindered isomerization of the azobenzene units [19–20]. The length and electronic nature of the spacer units control the distance from the surface and define the electronic coupling with the metal surface [11,18]. With increasing π-conjugation from the azobenzene into the platform, and thus coupling to the gold surface, the activation barrier drops to almost zero (≈8 kJ mol^−1^) and the frequency factors (log A) become negative [11]. Vanishing barriers and low frequency factors are typical for non-adiabatic reactions [9]. The mechanism was elucidated as a singlet–triplet–singlet spin change process, which is forbidden in solution but mediated by coupling to the conduction band of the bulk gold. We are now exploring scope and limitations of this peculiar spin catalysis. To investigate if the reverse isomerization process from the *trans* to the *cis*-configuration would also be accelerated, and to further scrutinize the coupling effects, we prepared analogous diazocine systems. Diazocines are bridged azobenzenes [21]. Imposed by the ring strain of the central eight-membered ring, the *cis*-configuration (boat conformation) is more stable than the *trans*-isomer (twist conformation). Upon irradiation with ≈400 nm the *cis*-form switches to the *trans*-isomer, and irradiation with ≈500 nm or heating leads back to the *cis*-form [22]. Hence, the diazocines are quasi reversed azobenzenes that are more stable in their *trans*-configurations [23].

**Figure 1:**
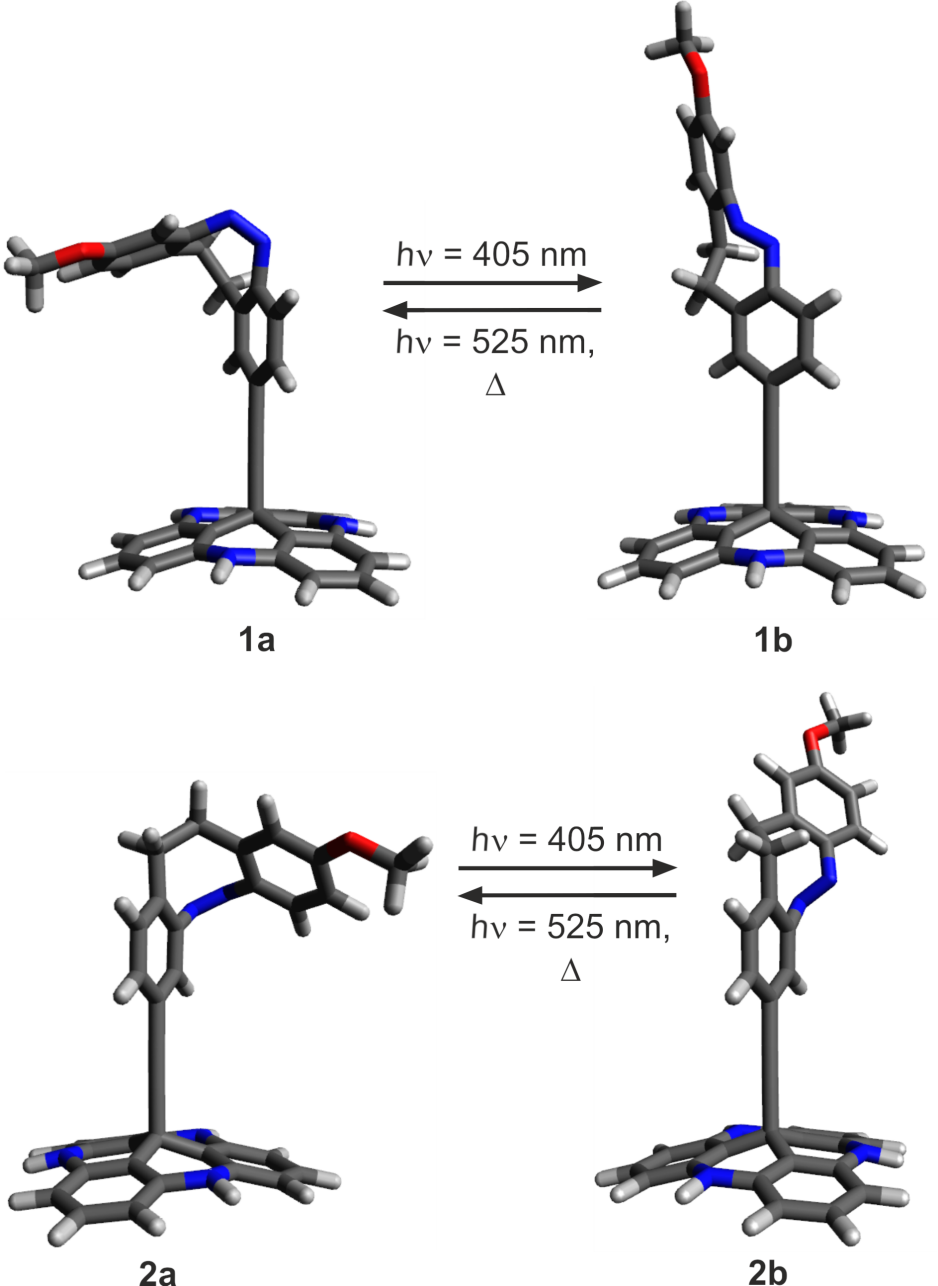
Structures of diazocine platform molecules (diazocine-TATAs) **1** and **2** in *cis* (**1a**, **2a**) and *trans*-configuration (**1b**, **2b**) (octyl side chains are replaced by protons for simplification). The *cis*-diazocines (**1a**, **2a**) isomerize upon irradiation with 405 nm to the metastable *trans*-diazocines (**1b**, **2b**) and with 525 nm or thermally back to the *cis*-diazocines (**1a**, **2a**).

To investigate the electronic coupling effects, we synthesized two diazocine derivatized TATA platforms with ethynyl spacers (diazocine-TATAs). In compound **1** the diazocine is connected to the platform with the ethynyl group in *para*-position to the azo group, providing a full π-conjugation path of the N=N unit through the ethynyl spacer into the platform. Diazocine-TATA **2** is connected in *meta*-position and thus interrupting conjugation [24–25]. Both diazocine-TATAs are equipped with methoxy groups, which serve as “reporter units” indicating the configuration of the molecules on metal surfaces [15]. In **1** the OMe group is attached *para* and in **2** the methoxy group is *meta* with respect to the azo group. Model calculations predict that the C_phenyl_–O bonds in the *cis*-isomers thus are parallel, and in the *trans*-isomers orthogonal to the surface (Figure 1). Previous investigations have shown that IRRAS (infrared absorption reflection spectroscopy) in combination with the surface selection rules (stretching mode orthogonal to the surface→high intensity, parallel to the surface→low intensity) is a suitable method to determine the configuration and to measure kinetics on surfaces [15]. The C–O stretching frequencies proved to be ideal reporter signals to determine the configuration and to measure kinetics in monolayers of azo-TATAs on surfaces.

## Results and Discussion

To obtain information on preferred conformations of **1** and **2** in their *cis* and *trans*-configurations and to predict thermodynamic and kinetic stabilities, we performed DFT calculations at the M06-2X/def2-TZVP level of theory (Table 1, for details see Supporting Information File 1, chapter VI). As expected for diazocine-based molecules our calculations predict the *cis* configuration for both compounds as the thermodynamically most stable isomers. For the corresponding *trans*-configuration two different conformations were found: the twist and the chair structures. The twist conformation is about 2.5 kcal mol^−1^ more stable than the chair conformation. Our calculations predict reaction barriers (*trans*-twist→*cis*-boat) for both compounds of approximately 23 kcal mol^−1^ (96 kJ mol^−1^). Obviously, the TATA platform and the ethynyl spacer have only marginal effects on the isomerization process. Hence, the diazocines **1** and **2** are ideal candidates to investigate the effect of bulk gold as a function of electronic coupling (conjugation) of the azo unit to gold.

**Table 1:** Calculated quantum chemical energies *E*_rel_ (M06-2X/def2-TZVP) of the twist and chair conformation of the *trans-*configuration of *para*-ethynyl-substituted diazocine **1b** (*para*-diazocine), and *meta*-diazocine **2b**, relative to the boat conformation of the *cis*-isomers **1a** and **2a**. Δ*H^≠^* are the calculated reaction barriers (*trans*-twist→*cis*- boat). All energies are given in kcal mol^−1^.

	*E*_rel_ *trans* twist	*E*_rel_ *trans* chair	Δ*H^≠^*

*para*-diazocine **1**	7.9	10.6	22.6
*meta*-diazocine **2**	8.0	10.3	23.0

**Scheme 1:**
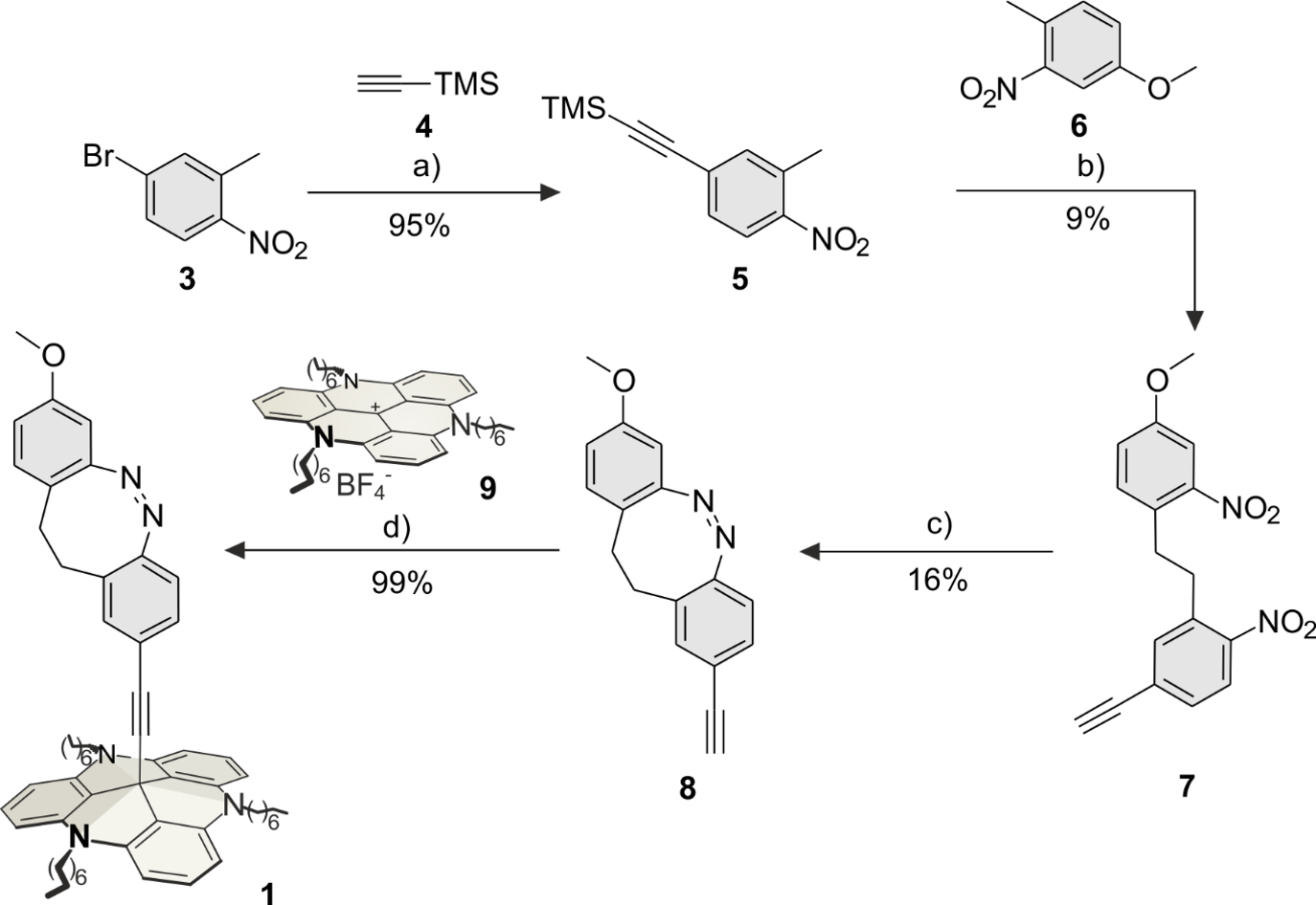
Synthesis route of *para*-diazocine platform molecule **1**. a) Pd(dppf)Cl_2_, Cu(I)I, Et_3_N, 1 h, 60 °C; b) 1: KO*t-*Bu, THF, 3 min, 0 °C, N_2_; 2: Br_2_, 5 min, 0 °C; c) 1: Ba(OH)_2_, Zn, EtOH, H_2_O, 4.75 h, reflux; 2: 0.1 M NaOH/MeOH, Cu(II)Cl_2_, 6 h, rt; d) KOH, THF, 3.5 h, reflux, N_2_.

The *para*-diazocine-TATA **1** was synthesized in a 5-step synthesis route (Scheme 1). Bromotoluene **3** was synthesized as described [26]. In a Sonogashira cross-coupling reaction acetylene-substituted toluene **5** was prepared from bromotoluene **3** with TMS-protected acetylene **4** (95%). The C–C bond formation of **5** and **6** to give dibenzoyl **7** was achieved with potassium butoxide and elemental bromine (9%) according to a literature procedure [27]. The *para*-ethynyldiazocine **8** was obtained by reduction of both nitro groups, followed by oxidation of the formed hydrazine (16%). The unprotected ethynyldiazocine **8** was deprotonated with potassium hydroxide and connected to the central carbon atom of the TATA platform **9** (synthesized according to Laursen and Krebs [28]) to obtain target *para*-diazocine mounted on the octyl-substituted TATA platform **1** (99%).

**Scheme 2:**
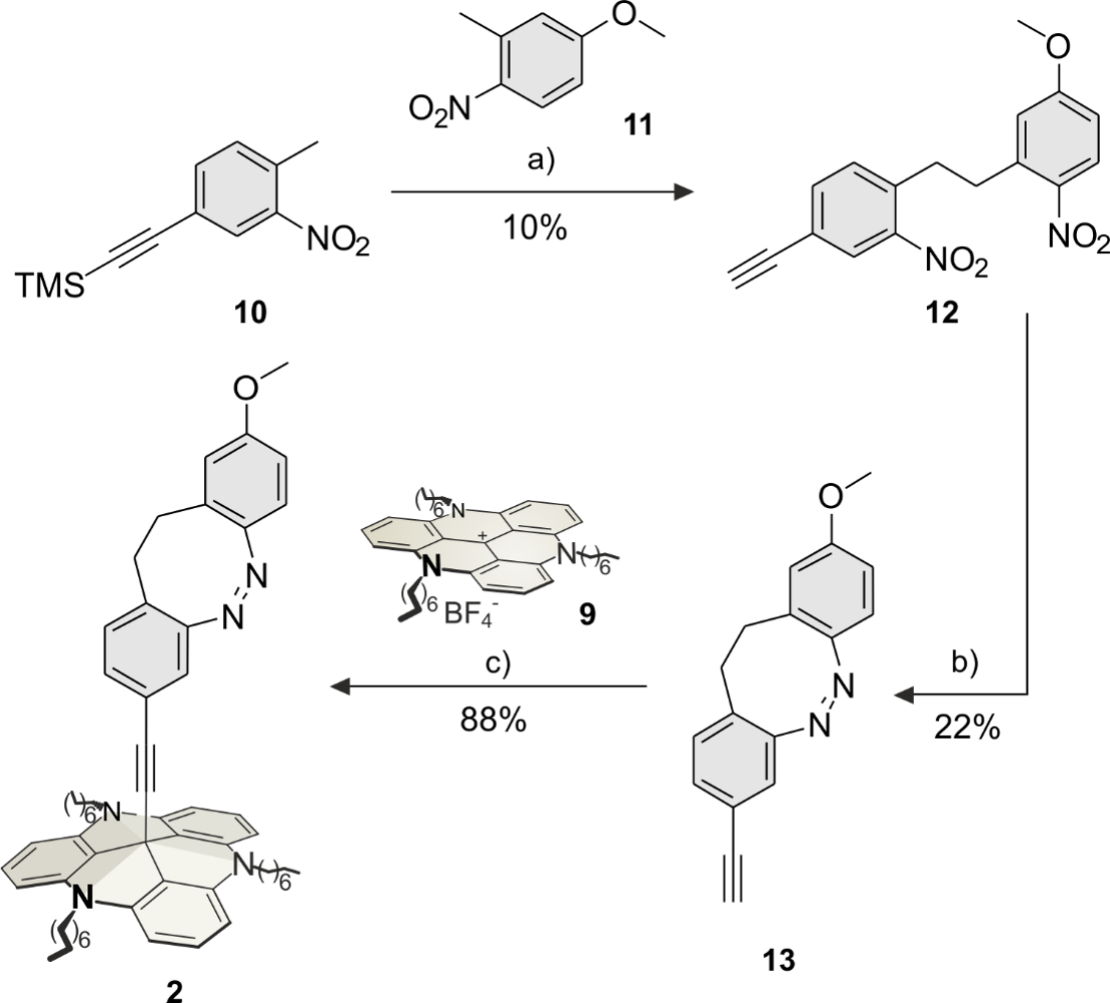
Synthesis route of *meta*-diazocine platform **2**. a) 1: KO*t-*Bu, THF, 3 min, 0 °C, N_2_; 2: Br_2_, 5 min, 0 °C, N_2_; b) 1: Ba(OH)_2_, Zn, EtOH, H_2_O, 4.75 h, reflux; 2: 0.1 M NaOH/MeOH, Cu(II)Cl_2_, 13 h, rt; c) KOH, THF, 2 h, reflux, N_2_.

The synthesis of the *meta-*diazocine platform molecule **2** was achieved in a 4-step synthesis route (Scheme 2). Nitrotoluene **10** was synthesized as described in literature [29]. The reaction of ethynyltoluene **10** with methoxytoluene **11** gave dibenzoyl **12** (10%) according to the same procedure as for dibenzoyl **7** (Scheme 1). Diazocine **13** was obtained by reduction and oxidation in moderate yields (22%). The reaction of diazocine **13** with the TATA ion **9** gave the target diazocine **2** (88%, Scheme 2).

**Figure 2:**
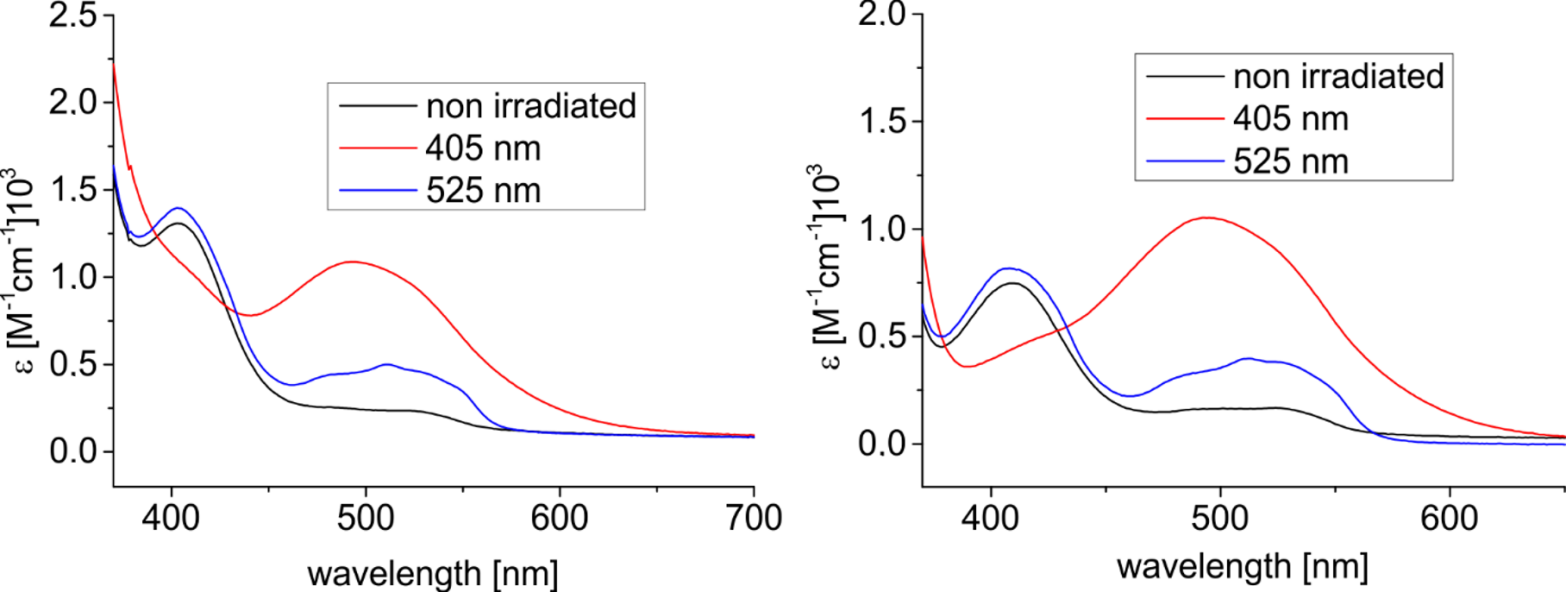
UV–vis spectra of **1** (left) and **2** (right) in THF at room temperature. Black: as synthesized, red: after irradiation with 405 nm, and blue: after irradiation with 525 nm.

The photophysical properties and the switching behavior of **1** and **2** were determined in solution (THF). The UV–vis spectra of **1** and **2** are shown before and after irradiation with 405 nm and 525 nm. Both diazocine-TATAs **1** and **2** exhibit similar UV spectra. The *n*→π* transition of *cis*-**1** appears at 403 nm and at 494 nm in the *trans-*isomer. The corresponding absorption maxima in diazocine-TATA **2** are 409 nm (*cis*) and 493 nm (*trans*) (Figure 2).

The photostationary states of **1** and **2** were determined in toluene-*d*_8_ by ^1^H NMR measurements (Table 2). Optimal wavelengths for the *cis*→*trans* isomerization are 405 nm (**1**: 53% *trans*, **2**: 65% *trans*). Back-isomerization to the *cis*-isomer with 525 nm is nearly quantitative. The half-lives (298 K) are similar for both systems (2.12 h for **1** and 2.32 h for **2**). The lack of conjugation between the azo function and the ethynyl spacer of **2** yields in a slightly higher half-life, which is in agreement with earlier results [11].

**Table 2:** Photostationary states (PSS) of *para*-diazocine-TATA **1** (2.05 mmol/L) and *meta*-diazocine-TATA **2** (2.27 mmol/L) upon irradiation with light of 405 nm, 525 nm and thermal isomerization half-life (*t*_1/2_) determined with ^1^H NMR spectroscopy (in deuterated toluene). The activation energies (*E*_a_) are calculated from the linear fit of an Arrhenius plot.

	*para*-diazocine **1**	*meta*-diazocine **2**

PSS (405 nm)	53% (*trans*)	65% (*trans*)
PSS (525 nm)	93% (*cis*)	93% (*cis*)
*t*_1/2_ (290.5 K)	5.27 h	5.76 h
*t*_1/2_ (298 K)	2.12 h	2.32 h
*t*_1/2_ (308 K)	0.69 h	0.73 h
E_A_ (kJ mol^−1^)	86.5	84.7

## STM Measurements

**Figure 3:**
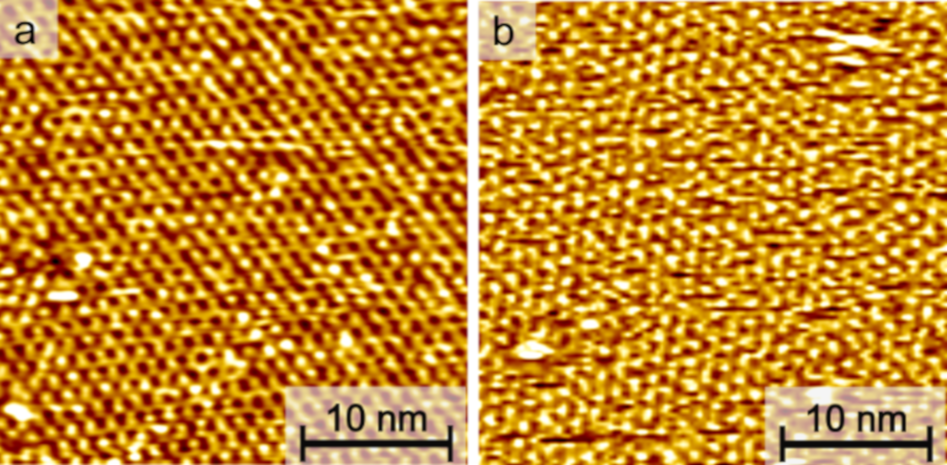
STM images (30 × 30 nm², *U*_bias_ = 0.3 V, *I*_t_ = 40 pA) of self-assembled monolayers of (a) compound **1** and (b) compound **2** on Au(111).

The adsorption behavior of the diazocine-TATA molecules on Au(111) surfaces was studied by STM at room temperature (Figure 3). Adlayers of both compounds show a hexagonally ordered superstructure with lattice constants of **1** and **2** being (12.2 ± 0.6) Å and (12.1 ± 0.5) Å, respectively. Additionally, two rotational domains with an angle of (15 ± 4)° are observed. Altogether these parameters are in good agreement with a (√19 × √19) *R*23.4° superstructure which has been also observed in previous STM investigations of TATA and azobenzene-TATA molecules with octyl ligands [18,20,30].

## Conclusion

In summary, we present the syntheses of two different diazocines mounted on TATA platforms (**1**, **2**). The photochemical switching between the stable *cis* and metastable *trans*-isomers was investigated. Upon irradiation with light of 405 nm diazocine-TATAs **1** and **2** convert to their *trans*-configurations in moderate to good yields. The metastable *trans*-isomers of **1** and **2** isomerize back to the *cis*-isomer with half-lives of 2.12 h and 2.32 h at 298 K. The *trans*→*cis* activation energies with 86.5 kJ mol^−1^ for **1** and with 84.7 kJ mol^−1^ for **2** are similar to the structurally related azobenzenes [11]. Both diazocine-TATAs form highly ordered monolayers on Au(111) surfaces. Further studies will include IRRAS measurements to determine the *trans*→*cis* isomerization kinetics on Au(111) surfaces.

## Experimental

For detailed experimental procedures, including NMR, UV–vis and MS spectra see Supporting Information File 1, chapters I–IV, for kinetic studies see Supporting Information File 1, chapter V.

## Supporting Information

File 1 Analytical methods, experimental procedures, NMR and UV spectra, kinetic studies and DFT calculations.
