# Supplementary material for: Diazocine-functionalized TATA platforms
Source: Beilstein J Org Chem. 2019 Jul 5;15:1485–90. doi: 10.3762/bjoc.15.150 (PMC6633206; doi:10.3762/bjoc.15.150)
Supplement: File 1 — Analytical methods, experimental procedures, NMR and UV spectra, kinetic studies and DFT calculations. [file Beilstein_J_Org_Chem-15-1485-s001.pdf]

BEILSTEIN JOURNAL OF ORGANIC CHEMISTRY

Supporting Information

for

Diazocine-functionalized TATA platforms

Roland Löw, Talina Rusch, Fynn Röhricht, Olaf Magnussen and Rainer Herges

Beilstein J. Org. Chem. **2019,** *15,* 1485–1490. doi:10.3762/bjoc.15.150

Analytical methods, experimental procedures, NMR and UV spectra, kinetic studies and DFT calculations

License and Terms: This is a supporting information file under the terms of the Creative Commons Attribution License (http://creativecommons.org/licenses/by/4.0). Please note that the reuse, redistribution and reproduction in particular requires that the authors and source are credited.
The license is subject to the *Beilstein Journal of Organic Chemistry* terms and conditions: (https://www.beilstein-journals.org/bjoc)

Table of Contents

I. Analytical equipment and methods

II. Experimental procedures

III. NMR spectra

IV. UV–vis absorption spectra

V. Kinetic studies in solution by ^{1}H NMR spectroscopy

VI. DFT Calculations

I. Analytical equipment and methods

NMR Spectroscopy

NMR spectra were measured in deuterated solvents (Deutero). All compounds were characterized using ^{1}H and ^{13}C NMR spectroscopy. The signals were assigned using 2D spectroscopy. For ^{1}H and ^{13}C NMR assignment we performed HSQC and HMBC. The degree of deuteration is given in parentheses. ^{1}H NMR spectra in reference to the following signals:

chloroform-d (99.8%): δ = 7.26 ppm. (s)

acetone-d_6 (99.5%): δ = 2.05 ppm. (quint.)

The signal multiplicities are abbreviated as follows:

s: singlet, d: doublet, dd: double doublet, t: triplet,

Measurements were performed by the following instruments:

Bruker CABAV 500neo (^{1}H NMR: 500 MHz, ^{13}C NMR: 125 MHz, ^{29}Si NMR: 99 MHz)

Bruker AV 600 (^{1}H NMR: 600 MHz, ^{13}C NMR: 150 MHz)

IR spectroscopy

Infrared spectra were measured on a Perkin-Elmer 1600 Series FT-IR spectrometer with an A531-G Golden-Gate-Diamond-ATR-unit. Signals were abbreviated with w, m, s and for weak, medium and strong intensities. Broad signals are additionally labeled with br.

Mass spectrometry

The high resolution (HR) mass spectra were measured with an APEX 3 FT-ICR with a 7.05 T magnet by co. Bruker Daltonics. Electron impact (EI).

Chromatrography stationary phases

For column chromatography purifications silica gel (Merck, particle size 0.040–0.063 mm) was used. R_f values were determined by thin layer chromatography onPolygram® Sil G/UV254 (Macherey-Nagel, 0.2 mm particle size).

II. Experimental procedures

II.1 1-Methyl-2-nitro-5-(2-(trimethylsilyl)ethynyl)benzene (5)

In triethylamine (dry, 80 mL) 4-bromo-2-methyl-1-nitrobenzol [1] **3** (6.00 g, 27.8 mmol), trimethylsilylacetylene (5.21 mL, 36.1 mmol), Pd(dppf)Cl_2 (1.02 g, 1.39 mmol, 5%) and cupper(I) iodide (530 mg, 2.78 mmol, 10%) were suspended under nitrogen atmosphere and stirred for 1 h at 60 °C. The reaction solution was filtered over celite and the solvent was removed under reduced pressure. The crude product was purified via column chromatography (silica gel, dichloromethane) to obtain a grey solid (6.14 g, 26.3 mmol, 95%).

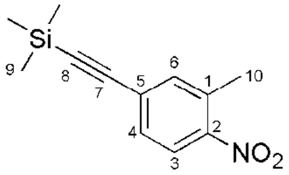

^{1}H NMR (500.1 MHz, $CDCl_3$, 298 K, TMS): δ = 7.93 (d, ^3J = 8.4 Hz, 1H, *H*-3), 7.43 (s, 1H, *H*-6), 7.40 (dd, ^3J = 8.5 Hz, ^4J = 1.6 Hz, 1H, *H*-4), 2.58 (s, 3H, *H*-10), 0.27 (s, 9H, *H*-9) ppm.

^{13}C NMR (125.8 MHz, $CDCl_3$, 298 K, CH_3Cl): δ = 148.43 (s, *C*-2), 136.21 (s, *C*-6), 133.98 (s, *C*-1), 130.30 (s, *C*-4), 128.47 (s, *C*-5), 124.93 (s, *C*-3), 102.91 (s, *C*-7), 99.44 (s, *C*-8), 20.53 (s, *C*-10), -0.12 (s, *C*-9) ppm.

^{29}Si NMR (99.4 MHz, $CDCl_3$, 298 K): δ = -16.99 ppm.

MS (EI, 70eV): m/z = 233.09 $[M]^+$.

IR (ATR): $\tilde{v}$ = 2957 (w), 2159 (w), 1601 (w), 1578 (w), 1515 (s), 1479 (w), 1348 (s), 1244 (s), 949 (m), 834 (vs), 756 (s), 698 (m), 663 (s), 450 (m) cm^{-1}.

m.p. = 76.5 °C.

HRMS (EI, 70 eV): m/z $[M]^+$ calcd. for $C_{12}H_{15}NO_2Si$: 233.08720; found: 233.08700.

II.2 4-Ethynyl-2-(4-methoxy-2-nitrophenethyl)-1-nitrobenzene (7)

In tetrahydrofurane (abs., 250 mL) 1-methyl-2-nitro-5-(2-(trimethylsilyl)ethynyl)benzene **5** (5.00 g, 21.4 mmol) and 4-methoxy-1-methyl-2-nitrobenzene (**6**, 2.97 mL, 21.4 mmol) were dissolved under nitrogen atmosphere and the solution was cooled to 0 °C. Potassium butoxide (3.40 g, 27.8 mmol) was added and stirred for 3 min before addition of bromine (1.42 mL, 27.8 mmol). After stirring for 5 min, the reaction was poured onto ice/water (500 mL). The solution was extracted with dichloromethane (3 × 300 mL) and the combined organic layers

were washed with saturated sodium thiosulfate solution and saturated sodium chloride solution and then dried over magnesium sulfate. The solvent was removed under reduced pressure and the crude product was purified via column chromatography (silica gel, cyclohexane/ethyl acetate, 2:1) to obtain a beige solid (615 mg, 1.89 mmol, 9%).

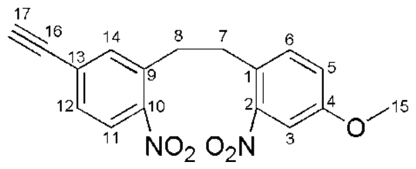

^{1}H NMR (500.1 MHz, $CDCl_3$, 298 K, TMS): δ = 7.92 (d, ^3J = 8.5 Hz, 1H, *H*-11), 7.55 (d, ^4J = 1.8 Hz, 1H, *H*-14), 7.50 (d, ^4J = 2.7 Hz, 1H, *H*-3), 7.47 (dd, ^3J = 8.4 Hz, ^4J = 1.8 Hz, 1H, *H*-12), 7.31 (d, ^3J = 8.5 Hz, 1H, *H*-6), 7.11 (dd, ^3J = 8.5 Hz, ^4J = 2.7 Hz, 1H, *H*-5), 3.87 (s, 3H, *H*-15), 3.30 (s, 1H, *H*-17), 3.23-3.13 (m, 4H, *H*-7, *H*-8) ppm.

^{13}C NMR (125.8 MHz, $CDCl_3$, 300 K, TMS): δ = 158.61 (s, *C*-4), 149.43 (s, *C*-2), 148.63 (s, *C*-10), 136.49 (s, *C*-9), 136.01 (s, *C*-14), 133.27 (s, *C*-6), 130.94 (s, *C*-12), 127.76 (s, *C*-1), 127.59 (s, *C*-13), 124.98 (s, *C*-11), 120.28 (s, *C*-5), 109.35 (s, *C*-3), 81.55 (s, *C*-17), 55.85 (s, *C*-15), 34.36 (s, *C*-8), 33.79 (s, *C*-7) ppm.

MS (EI, 70eV): m/z = 326.08 $[M]^+$.

IR (ATR): $\tilde{\nu}$ = 3286 (m), 2939 (br. w), 1602 (s), 1573 (w), 1485 (vs), 1461 (m), 1309 (w), 1278 (s), 1245 (vs), 1156 (w), 1107 (w), 1037 (m), 898 (w), 865 (w), 812 (m), 658 (w), 616 (w) cm^{-1}.

m.p. = 104.1 °C.

HRMS (EI, 70 eV): m/z $[M]^+$ calcd. for $C_{17}H_{14}N_2O_5$: 326.09027; found: 326.09052.

II.3 (*Z*)-2-Ethynyl-8-methoxy-11,12-dihydrodibenzo[*c*,*g*][1,2]-diazocine (8)

In ethanol (100 mL) 4-ethynyl-2-(4-methoxy-2-nitrophenethyl)-1-nitrobenzene (**7**, 400 mg, 1.23 mmol) was dissolved, an aqueous solution of barium hydroxide $[Ba(OH)_2 \cdot 8H_2O]$ (1.16 g, 3.68 mmol) in H_2O (40 mL) and zinc powder (1.29 g, 19.7 mmol) were added. The reaction was refluxed for 4.75 h. The reaction mixture was filtered through celite and the solvent was removed under reduced pressure. The crude product was dissolved in dichloromethane, filtered through celite and the solvent was removed under reduced pressure. The crude product was dissolved in 0.1 M methanolic NaOH solution (120 mL), $CuCl_2$ (6.60 mg, 49.1 µmol) was added and air was bubbled through the solution for 6 h at room temperature. The reaction was neutralized with 1 M hydrogen chloride solution and saturated sodium bicarbonate solution

(150 mL) was added. The aqueous layer was extracted with dichloromethane (3 × 150 mL) and the solvent was removed under reduced pressure. The crude product was purified via column chromatography (silica gel, cyclohexane/ethyl acetate, 2/1) to obtain an orange solid (51.0 mg, 194 µmol, 16%).

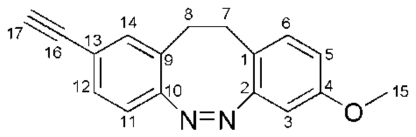

^{1}H NMR (500.1 MHz, acetone-d$_6$, 298 K, TMS): δ = 7.28 (dd, ^3J = 8.1 Hz, ^4J = 1.7 Hz, 1H, *H*-12), 7.20 (d, ^4J = 1.6 Hz, 1H, *H*-14), 6.97 (d, ^3J = 8.4 Hz, 1H, *H*-6), 6.83 (d, ^3J = 8.1 Hz, 1H, *H*-11), 6.61 (dd, ^3J = 8.4 Hz, ^4J = 2.6 Hz, 1H, *H*-5), 6.39 (d, ^4J = 2.7 Hz, 1H, *H*-3), 3.71 (s, 3H, *H*-15), 3.59 (s, 1H, *H*-17), 2.87-2.81 (m, 4H, *H*-7, *H*-8) ppm.

^{13}C NMR (125.8 MHz, acetone-d$_6$, 298 K, acetone): δ= 159.44 (s, *C*-4), 157.41 (s, *C*-2), 156.85 (s, *C*-10), 134.12 (s, *C*-14), 131.82 (s, *C*-6), 131.10 (s, *C*-12), 130.21 (s, *C*-9), 121.72 (s, *C*-13), 120.61 (s, *C*-1), 119.68 (s, *C*-11), 113.61 (s, *C*-5), 104.60 (s, *C*-3), 83.52 (s, *C*-16), 79.25 (s, *C*-17), 55.66 (s, *C*-15), 31.86 (s, *C*-8), 31.10 (s, *C*-7) ppm.

MS (EI, 70eV): m/z = 262.11 [M]$^+$.

IR (ATR)**:** ṽ = 3275 (m), 1605 (w), 1493 (s), 1393 (w), 1251 (m), 1143 (w), 1065 (w), 1030 (vs), 999 (s), 899 (w), 811 (vs), 671 (s), 616 (s), 509 (s) cm^{-1}.

m.p. = 133.8 °C.

HRMS (EI, 70 eV): m/z [M]+ calcd. for $C_{17}H_{14}N_2O$: 262.11061; found: 262.11019.

II.4 (*Z*)-12c-(8-Methoxy-11,12-dihydrodibenzo[c,g][(1,2]diazocin-2-yl)ethynyl-4,8,12-tri-*n*-octyl-4,8,12-triazatriangulene (1)

In tetrahydrofurane (abs., 30 mL) (*Z*)-2-ethynyl-8-methoxy-11,12-dihydrodibenzo(*c*,*g*)(1,2)-diazocine (**8**, 46.0 mg, 175 µmol), octyl-TATA-BF$_4$ [2] **9** (161 mg, 228 µmol) and powdered potassium hydroxide (105 mg, 1.87 mmol) were suspended under nitrogen atmosphere and refluxed for 3.5 h. The reaction was poured onto saturated sodium chloride solution (20 mL) and the aqueous layer was extracted with diethyl ether (3 × 30 mL). The combined organic layers were dried over magnesium sulfate and the solvent was removed under reduced pressure. The crude product was purified via column chromatography (aluminium oxide, basic, diethylether) to obtain an orange solid (153 mg, 174 µmol, 99%).

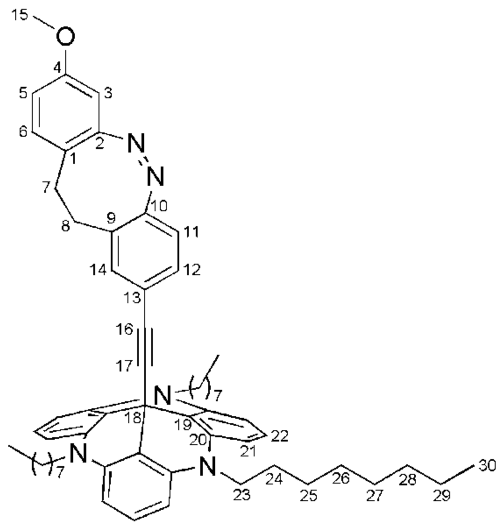

^{1}H NMR (500.1 MHz, acetone-d$_6$, 298 K, TMS): δ = 7.18 (t, ^{3}J = 8.3 Hz, 3H, *H*-22), 6.85-6.80 (m, 2H, *H*-6, *H*-12), 6.72 (d, ^{4}J = 1.6 Hz, 1H, *H*-14), 6.65-6.60 (m, 7H, *H*-11, *H*-21), 6.47 (dd, ^{3}J = 8.4 Hz, ^{4}J = 2.7 Hz, 1H, *H*-5), 6.26 (d, ^{4}J = 2.6 Hz, 1H, *H*-3), 3.99-3.93 (ps. t, 6H, *H*-23), 3.63 (s, 3H, *H*-15), 2.76-2.62 (m, 4H, *H*-7, *H*-8), 1.84-1.77 (ps. sechst., 6H, *H*-24), 1.52-1.45 (ps. pent., 6H, *H*-25), 1.41-1.21 (m, 24H, *H*-26, *H*-27, *H*-28, *H*-29), 0.90-0.85 (ps. t, 9H, *H*-30) ppm.

^{13}C NMR (125.8 MHz, acetone-d$_6$, 298 K, acetone): δ= 159.31 (s, *C*-4), 157.40 (s, *C*-2), 156.04 (s, *C*-10), 141.34 (s, *C*-20), 133.14 (s, *C*-14), 131.61 (s, *C*-6), 130.32 (s, *C*-12), 129.81 (s, *C*-9), 129.38 (s, *C*-22), 123.01 (s, *C*-13), 120.62 (s, *C*-1), 119.25 (s, *C*-11), 113.28 (s, *C*-5), 110.82 (s, *C*-19), 106.03 (s, *C*-21), 104.50 (s, *C*-3), 94.77 (s, *C*-17), 83.72 (s, *C*-16), 55.56 (s, *C*-15), 46.72 (s, *C*-23), 32.58 (s, *C*-29), 31.77 (s, *C*-8), 31.01 (s, *C*-7), 30.11 (s, C-26), 30.06 (s, C-27), 27.43 (s, *C*-25), 26.62 (s, *C*-24), 23.33 (s, *C*-28), 14.40 (s, *C*-30) ppm.

MS (MALDI-TOF, Cl-CCA): m/z = 879.6 $[M]^+$.

IR (ATR)**:** ṽ = 2924 (m), 2852 (m), 1614 (vs), 1579 (vs), 1482 (vs), 1457 (vs), 1393 (vs), 1267 (m), 1242 (s), 1167 (s), 1145 (m), 1036 (w), 895 (w), 809 (w), 772 (m), 748 (m), 726 (s), 657 (w) cm^{-1}.

II.5 4-Ethynyl-1-(5-methoxy-2-nitrophenethyl)-2-nitrobenzene (12)

In tetrahydrofurane (abs., 280 mL) 1-methyl-2-nitro-4-(2-(trimethylsilyl)ethynyl)-benzene (**10**) [3] (6.00 g, 25.7 mmol) and 4-methoxy-2-methyl-1-nitrobenzene (**11**, 6.45 g, 30.9 mmol) were dissolved under nitrogen atmosphere and cooled to 0 °C. Potassium butoxide (4.40 g, 36.0 mmol) was added and stirred for 3 min before addition of bromine (1.84 mL, 36.0 mmol). After stirring for 5 min, the reaction was poured onto ice/water (500 mL). The solution was

extracted with dichloromethane (3 × 300 mL) and the combined organic layers were washed with saturated sodium thiosulfate solution and saturated sodium chloride solution and then dried over magnesium sulfate. The solvent was removed under reduced pressure and the crude product was purified via column chromatography (silica gel, cyclohexane/ethyl acetate, 2/1) to obtain a beige solid (817 mg, 2.50 mmol, 10%).

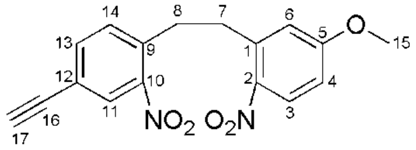

^{1}H NMR (600.1 MHz, $CDCl_3$, 298 K, TMS): δ = 8.10 (d, ^3J = 9.0 Hz, 1H, *H*-3), 8.06 (d, ^4J = 1.6 Hz, 1H, *H*-11), 7.63 (dd, ^3J = 8.0 Hz, 1H, *H*-13), 7.44 (d, ^3J = 7.9 Hz, 1H, *H*-14), 6.85 (dd, ^3J = 9.1 Hz, ^4J = 2.8 Hz, 1H, *H*-4), 6.83 (d, ^4J = 2.8 Hz, 1H, *H*-6), 3.88 (s, 3H, *H*-15), 3.30-3.21 (m, 4H, *H*-7, *H*-8), 3.18 (s, 1H, *H*-17) ppm.

^{13}C NMR (150.9 MHz, $CDCl_3$, 298 K, TMS): δ = 163.40 (s, *C*-5), 149.01 (s, *C*-10), 141.85 (s, *C*-2), 139.20 (s, *C*-1), 136.67 (s, *C*-9), 136.38 (s, *C*-13), 132.67 (s, *C*-14), 128.24 (s, *C*-11), 127.97 (s, *C*-3), 121.96 (s, *C*-12), 116.85 (s, *C*-4), 112.97 (s, *C*-6), 81.00 (s, *C*-16), 79.56 (s, *C*-17), 55.89 (s, *C*-15), 35.37 (s, *C*-7), 34.14 (s, *C*-8) ppm.

MS (EI, 70eV): m/z = 326.06 $[M]^+$.

$]^+$. **IR** (ATR): $\tilde{v}$ = 3274 (m), 1601 (m), 1586 (m), 1548 (w), 1521 (s), 1500 (s), 1455 (w), 1343 (m), 1327 (vs), 1298 (w), 1258 (vs), 1211 (m), 1077 /m), 1069 (m), 1030 (m), 896 (m), 870 (m), 831 (m), 804 (m), 762 (m), 699 (m) cm^{-1}.

m.p. = 127.1 °C.

HRMS (EI, 70 eV): m/z $[M]^+$ calcd. for $C_{17}H_{14}N_2O_5$: 326.09027; found: 326.09068.

II.6 (*Z*)-8-Ethynyl-2-methoxy-11,12-dihydrodibenzo[*c*,*g*][1,2]-diazocine (13)

In ethanol (80 mL) 4-Ethynyl-1-(5-methoxy-2-nitrophenethyl)-2-nitrobenzene (**12**, 396 mg, 1.21 mmol) was dissolved, an aqueous solution of barium hydroxide $[Ba(OH)_2 \cdot 8H_2O]$ (1.15 g, 3.64 mmol) in H_2O (34 mL) and zinc powder (1.27 g, 19.4 mmol) were added. The reaction was refluxed for 4.75 h. The reaction mixture was filtered through celite and the solvent was removed under reduced pressure. The crude product was dissolved in dichloromethane (50 mL), filtered through celite and the solvent was removed under reduced pressure. The crude product was dissolved in 0.1 M sodium hydroxide solution in methanol (120 mL), $CuCl_2$ (6.5 mg, 48 µmol) was added and air was bubbled through the solution for 13 h at room

temperature. The reaction was neutralized with 1 M hydrogen chloride solution and saturated sodium bicarbonate solution (200 mL) was added. The aqueous layer was extracted with dichloromethane and the solvent was removed under reduced pressure. The crude product was purified via column chromatography (silica gel, cyclohexane/ethyl acetate, 2:1) to obtain an orange solid (69.0 mg, 263 µmol, 22%).

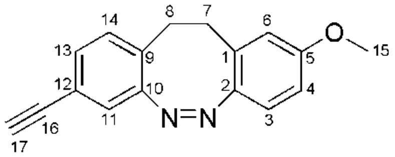

^{1}H NMR (500.1 MHz, $CDCl_3$, 298 K, TMS): δ = 7.15 (dd, ^{3}J = 7.9 Hz, ^{4}J = 1.6 Hz, 1H, *H*-13), 6.96 (d, ^{3}J = 7.8 Hz, 1H, *H*-14), 6.94 (d, ^{4}J = 1.6 Hz, 1H, *H*-11), 6.83 (d, ^{3}J = 8.7 Hz, 1H, *H*-3), 6.69 (dd, ^{3}J = 8.6 Hz, ^{4}J = 2.6 Hz, 1H, *H*-4), 6.49 (d, ^{4}J = 2.6 Hz, 1H, *H*-6), 3.71 (s, 3H, *H*-15), 3.03 (s, 1H, *H*-17), 2.85 (m, 4H, *H*-7, *H*-8) ppm.

^{13}C NMR (125.8 MHz, $CDCl_3$, 298 K, TMS): δ = 158.36 (s, *C*-5), 155.27 (s, *C*-10), 148.95 (s, *C*-2), 130.61 (s, *C*-13), 129.64 (s, *C*-14), 129.46 (s, *C*-9), 129.15 (s, *C*-1), 122.37 (s, *C*-11), 120.94 (s, *C*-3), 120.54 (s, *C*-12), 114.77 (s, *C*-6), 111.98 (s, *C*-4), 82.71 (s, *C*-16), 77.62 (s, C-17), 55.27 (s, *C*-15), 32.08 (s, *C*-7), 31.47 (s, *C*-8) ppm.

MS (EI, 70eV): m/z = 262.11 $[M]^+$.

IR (ATR): $\tilde{\nu}$ = 3265 (m), 2919 (br. w), 2851 (w), 1609 (w), 1575 (m), 1483 (s), 1464 (m), 1427 (m), 1308 (m), 1259 (vs), 1154 (m), 1110 (m), 1040 (m), 864 (m), 819 (m), 798 (vs), 703 (m), 613 (s), 586 (s) cm^{-1}.

m.p. = 112.1 °C.

HRMS (EI, 70 eV): m/z [M]+ calcd. for $C_{17}H_{14}N_2O$: 262.11061; found: 262.11016.

II.7 (*Z*)-12c-(9-Methoxy-11,12-dihydrodibenzo[c,g][1,2]diazocin-3-yl)ethynyl-4,8,12-tri-*n*-octyl-4,8,12-triazatriangulene (2)

In tetrahydrofurane (abs., 10 mL) (*Z*)-8-Ethynyl-2-methoxy-11,12-dihydrodibenzo(*c*,*g*)(1,2)-diazocine (**13**, 20.0 mg, 76.2 µmol), octyl-TATA-BF_4 [2] **9** (64.6 mg, 91.5 µmol) and powdered potassium hydroxide (34.2 mg, 610 µmol) were suspended under nitrogen atmosphere and refluxed for 2 h. The reaction was poured onto saturated sodium chloride solution (30 mL) and extracted with diethylether (3 × 25 mL). The combined organic layers were dried over magnesium sulfate and the solvent was removed under reduced pressure. The crude product

was purified via column chromatography (aluminium oxide, basic, diethylether) to obtain an orange solid (59.0 mg, 67.0 µmol, 88%).

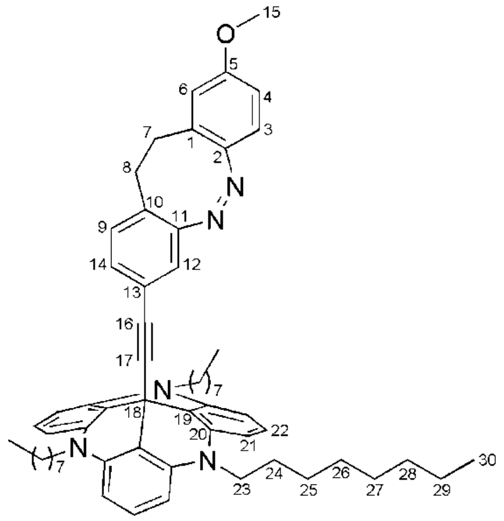

^{1}H NMR (500.1 MHz, acetone-d$_6$, 298 K, TMS): δ= 7.18 (t, ^{3}J = 8.24 Hz, 3H, *H*-22), 6.89 (d, ^{3}J = 8.0 Hz, 1H, *H*-9), 6.71 (dd, ^{3}J = 8.0 Hz, ^{4}J= 1.7 Hz, 1H, *H*-14), 6.68 (d, ^{3}J = 8.5 Hz, 1H, *H*-3), 6.65-6.60 (m, 7H, *H*-4, *H*-21), 6.52 (d, ^{4}J = 2.6 Hz, 1H, *H*-6), 6.44 (d, ^{4}J = 1.6 Hz, 1H, *H*-12), 3.99-3.93 (ps. t, 6H, *H*-23), 3.63 (s, 3H, *H*-15), 2.78-2.69 (m, 4H, *H*-7, *H*-8), 1.85-1.76 (ps. pent., 6H, *H*-24), 1.52-1.44 (ps. pent., 6H, *H*-25), 1.41-1.22 (m, 24H, *H*-26, *H*-27, *H*-28, *H*-29), 0.90-0.85 (ps. t, 9H, *H*-30) ppm.

^{13}C NMR (125.8 MHz, acetone-d$_6$, 298 K, acetone): δ = 159.25 (s, *C*-5), 156.54 (s, *C*-11), 150.00 (s, *C*-2), 141.36 (s, *C*-20), 130.52 (s, *C*-9), 130.38 (s, *C*-14), 130.27 (s, *C*-1), 129.44 (s, *C*-10), 129.38 (s, *C*-22), 122.89 (s, *C*-13), 121.52 (s, *C*-12), 121.15 (s, *C*-3), 115.65 (s, *C*-6), 112.54 (s, *C*-4), 110.86 (s, *C*-19), 106.05 (s, *C*-21), 95.09 (s, *C*-17), 83.45 (s, *C*-16), 55.48 (s, *C*-15), 46.67 (s, *C*-23), 32.57 (s, *C*-29), 32.38 (s, *C*-7), 31.73 (s, *C*-8), 30.10 (s, *C*-26), 30.05 (s, *C*-27), 27.43 (s, *C*-25), 26.65 (s, *C*-24), 23.32 (s, *C*-28), 14.40 (s, *C*-30) ppm.

MS (MALDI-TOF): m/z = 879.6 [M]$^{+}$.

IR (ATR): $\tilde{\nu}$ = 2924 (s), 2852 (m), 1614 (s), 1579 (vs), 1482 (vs), 1457 (vs), 1393 (vs), 1374 (m), 1243 (s), 1167 (s), 1039 (w), 911 (w), 807 (w), 772 (m), 750 (s), 724 (s), 657 (w) cm^{-1}.

III. NMR Spectra

III.1 1-Methyl-2-nitro-5-(2-(trimethylsilyl)ethynyl)benzene

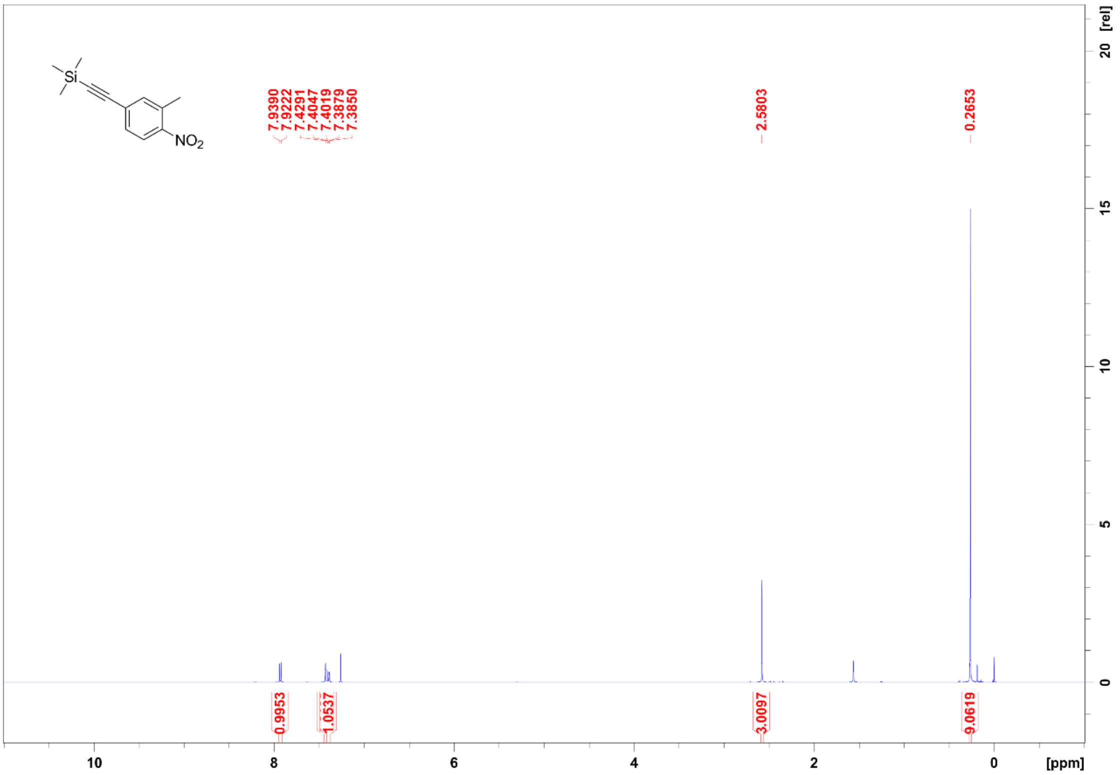

Figure S1. ^{1}H NMR spectrum (500.1 MHz, $CDCl_3$) of compound **5**.

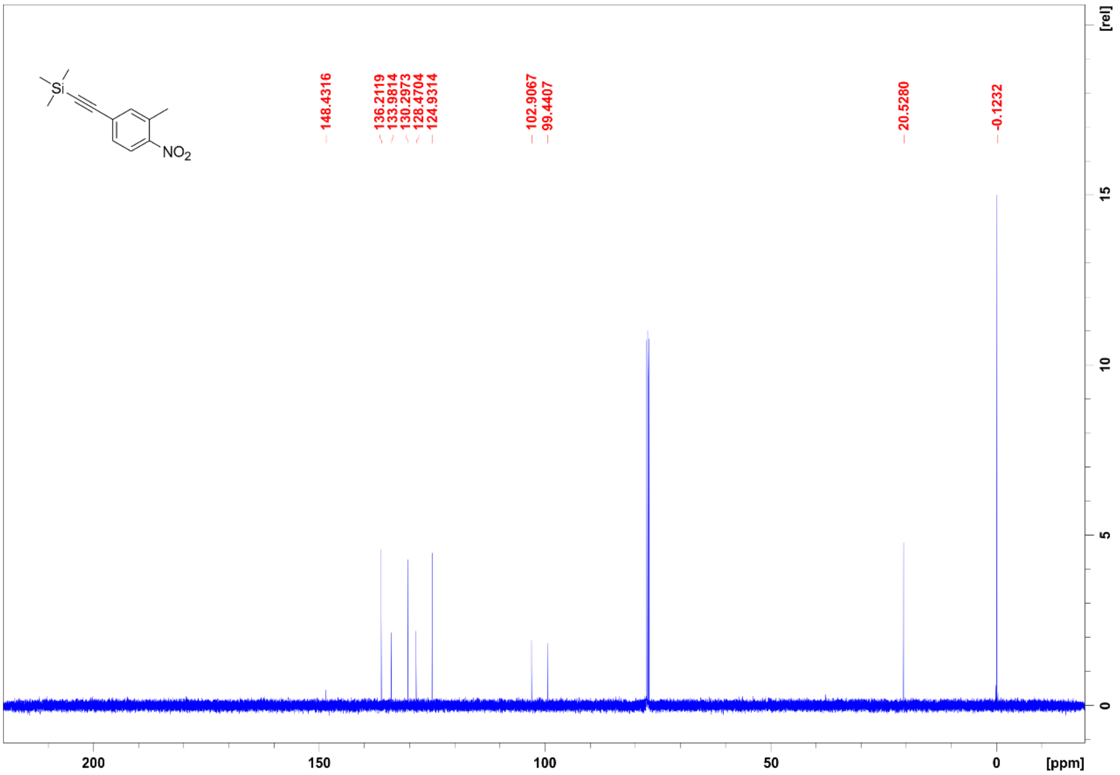

Figure S2. ^{13}C NMR spectrum (125.8 MHz, $CDCl_3$) of compound **5**.

III.2 4-Ethynyl-2-(4-methoxy-2-nitrophenethyl)-1-nitrobenzene

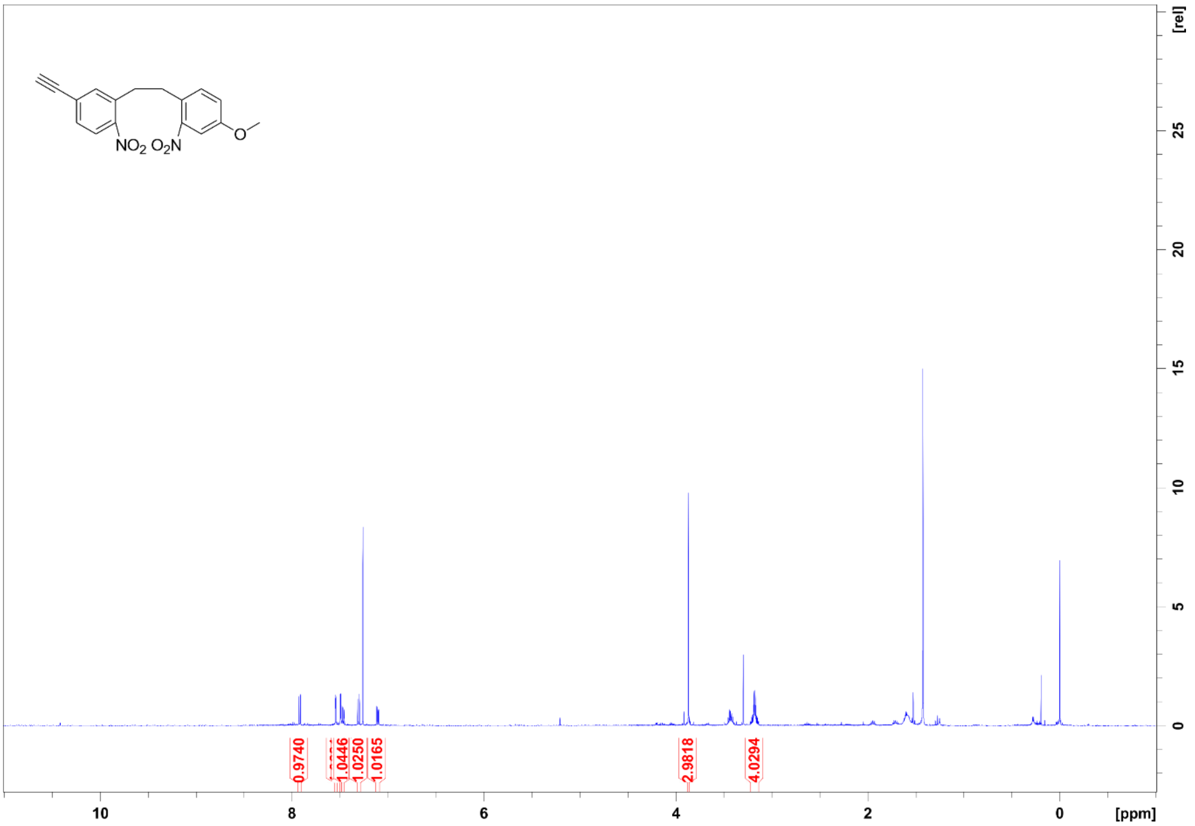

Figure S3. ^{1}H NMR spectrum (500.1 MHz, $CDCl_3$) of compound **7**.

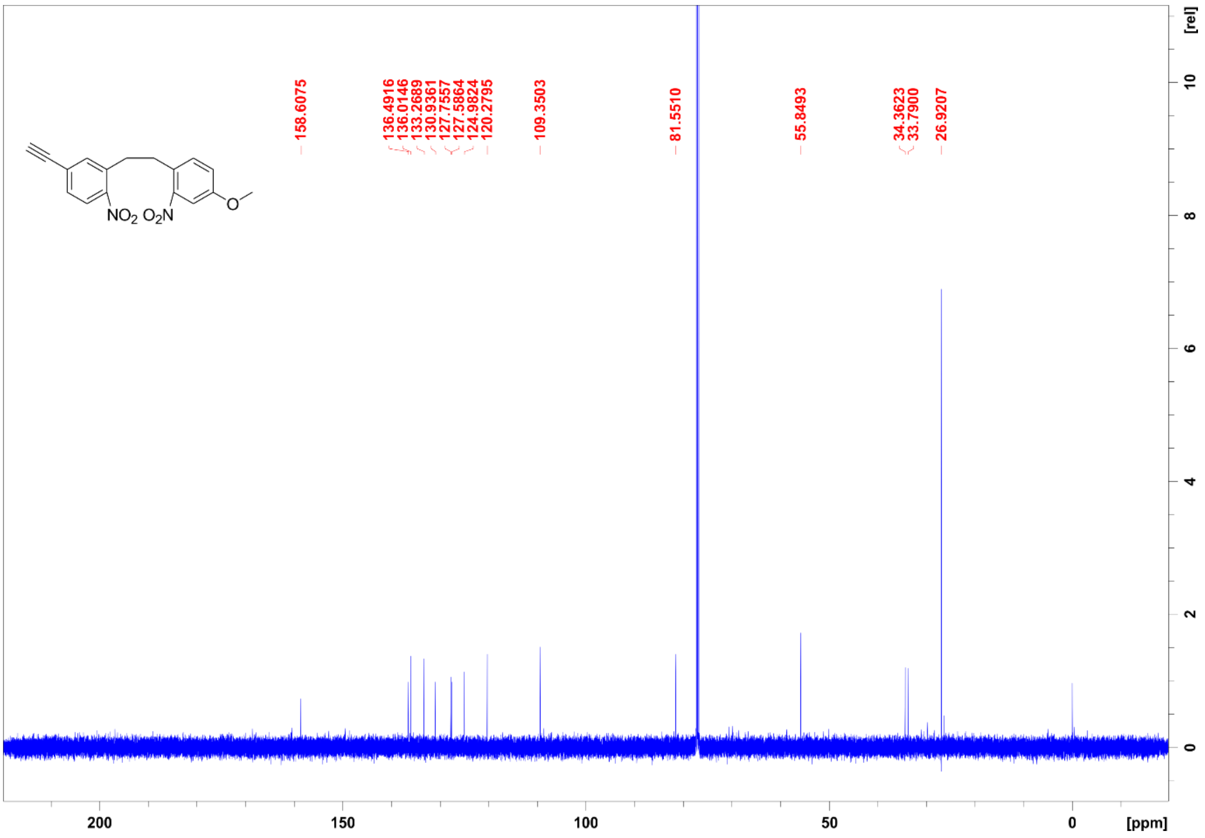

Figure S4. ^{13}C NMR spectrum (125.8 MHz, $CDCl_3$) of compound **7**.

III.3 (*Z*)-2-Ethynyl-8-methoxy-11,12-dihydrodibenzo[*c*,*g*][1,2]-diazocine

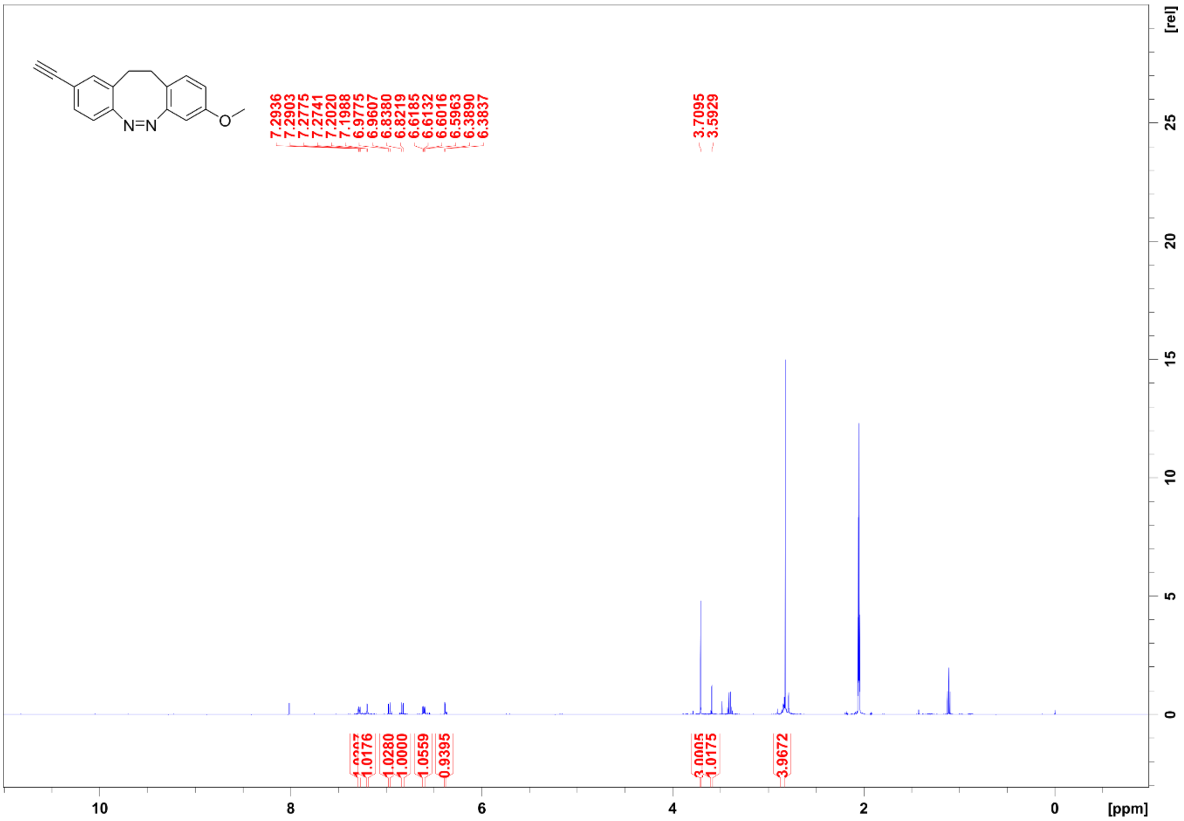

Figure S5. ^{1}H NMR spectrum (500.1 MHz, acetone-d_6) of compound **8**.

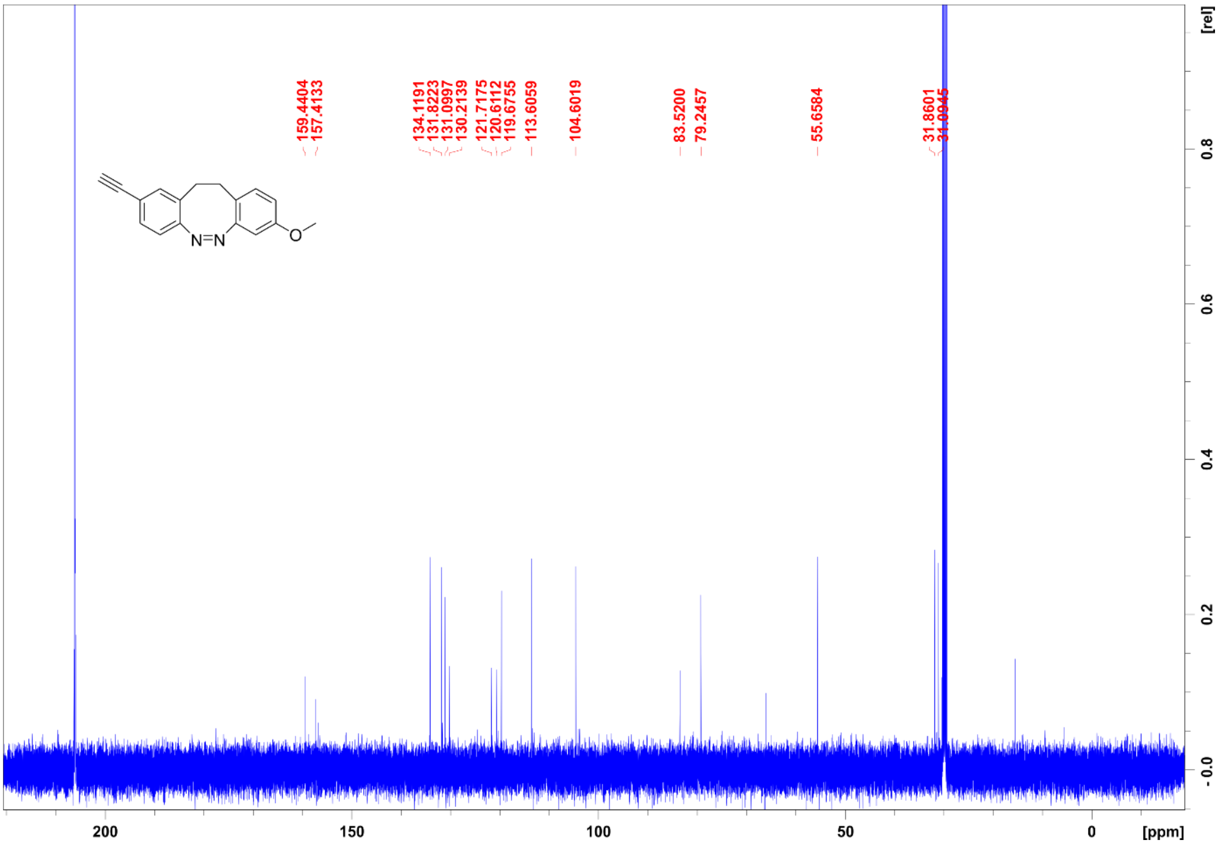

Figure S6. ^{13}C NMR spectrum (125.8 MHz, acetone-d_6) of compound **8**.

III.4 (*Z*)-12c-(8-Methoxy-11,12-dihydrodibenzo[c,g][1,2]diazocin-2-yl)ethynyl-4,8,12-tri-*n*-octyl-4,8,12-triazatriangulene

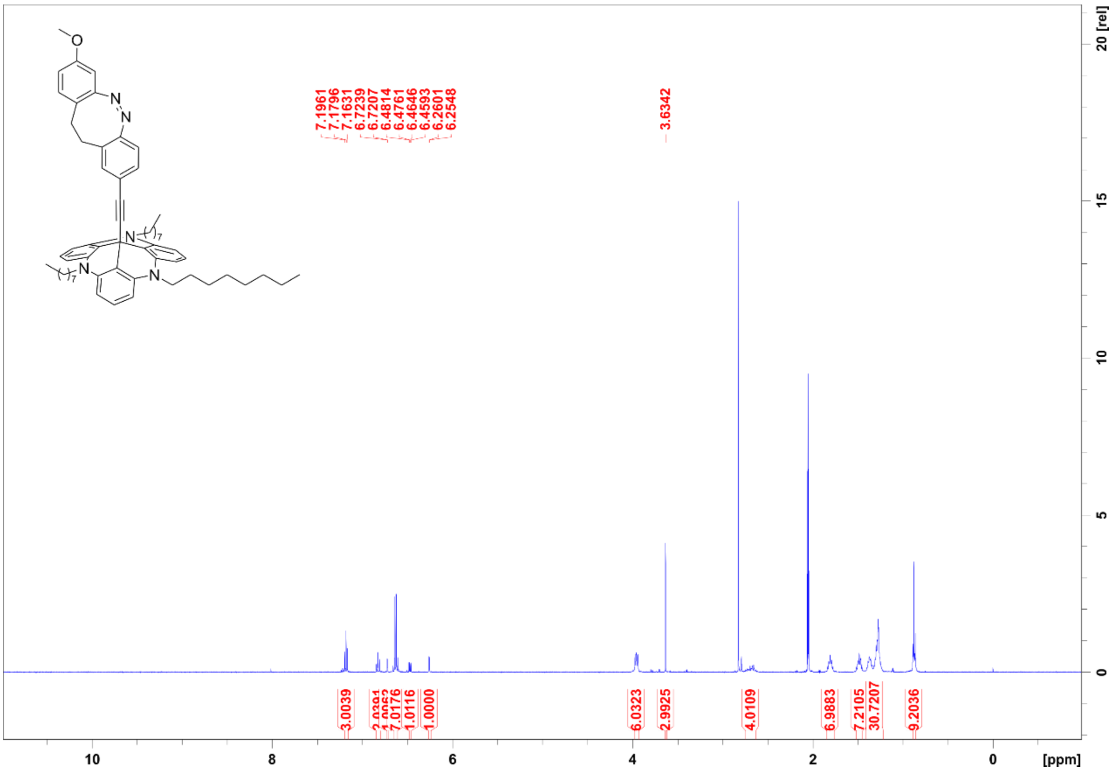

Figure S7. ^{1}H NMR spectrum (500.1 MHz, acetone-d_6) of compound **1**.

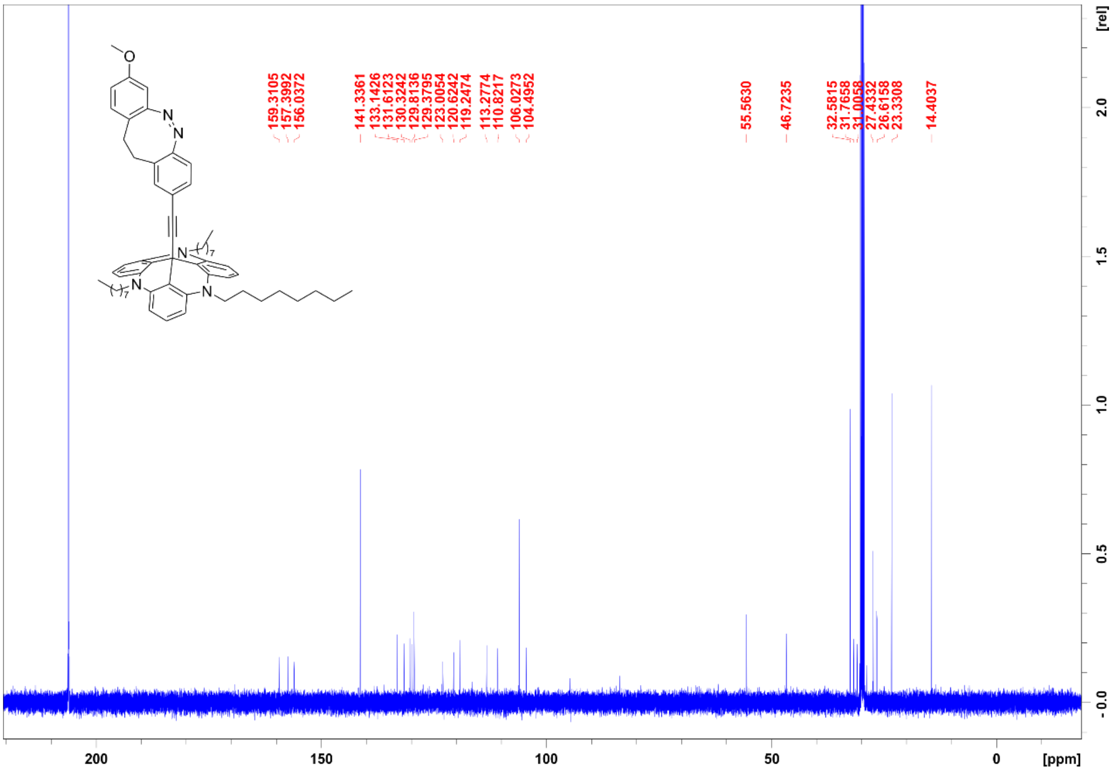

Figure S8. ^{13}C NMR spectrum (125.8 MHz, acetone-d_6) of compound **1**.

III.5 4-Ethynyl-1-(5-methoxy-2-nitrophenethyl)-2-nitrobenzene

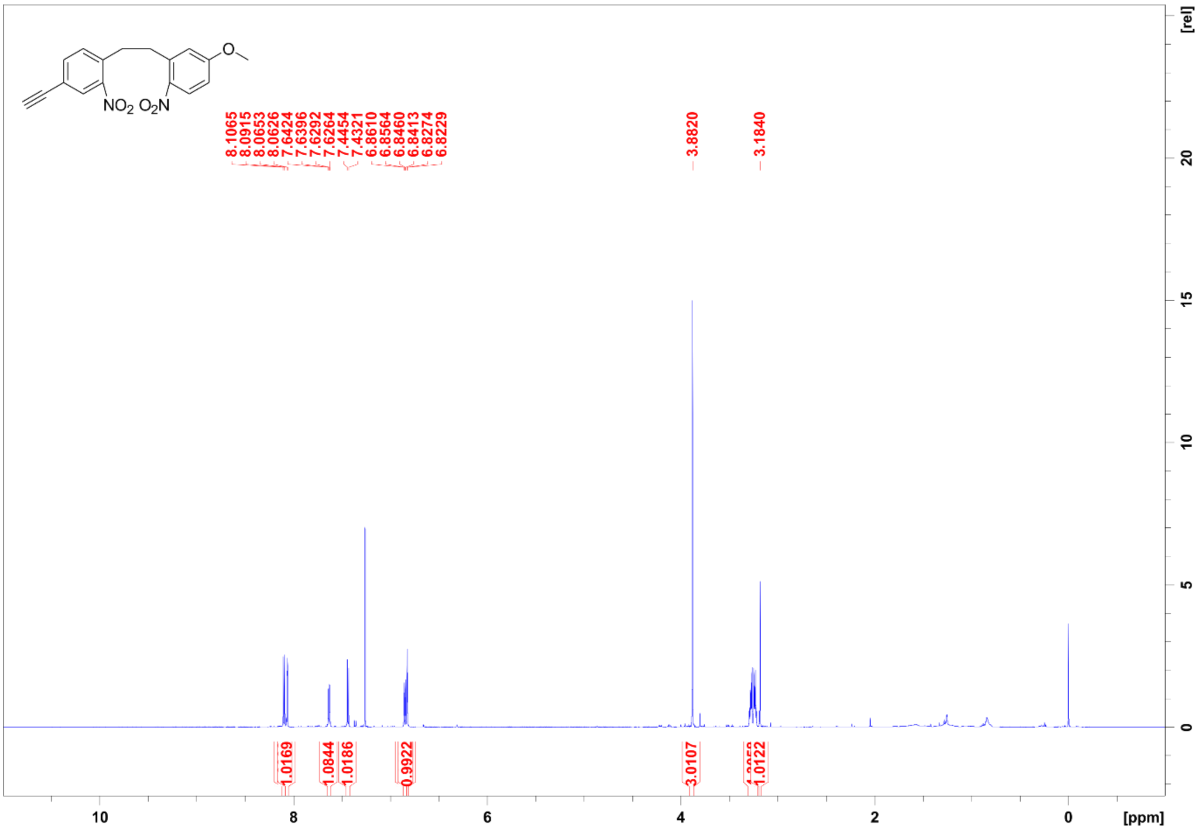

Figure S9. ^{1}H NMR spectrum (600.1 MHz, $CDCl_3$) of compound **12**.

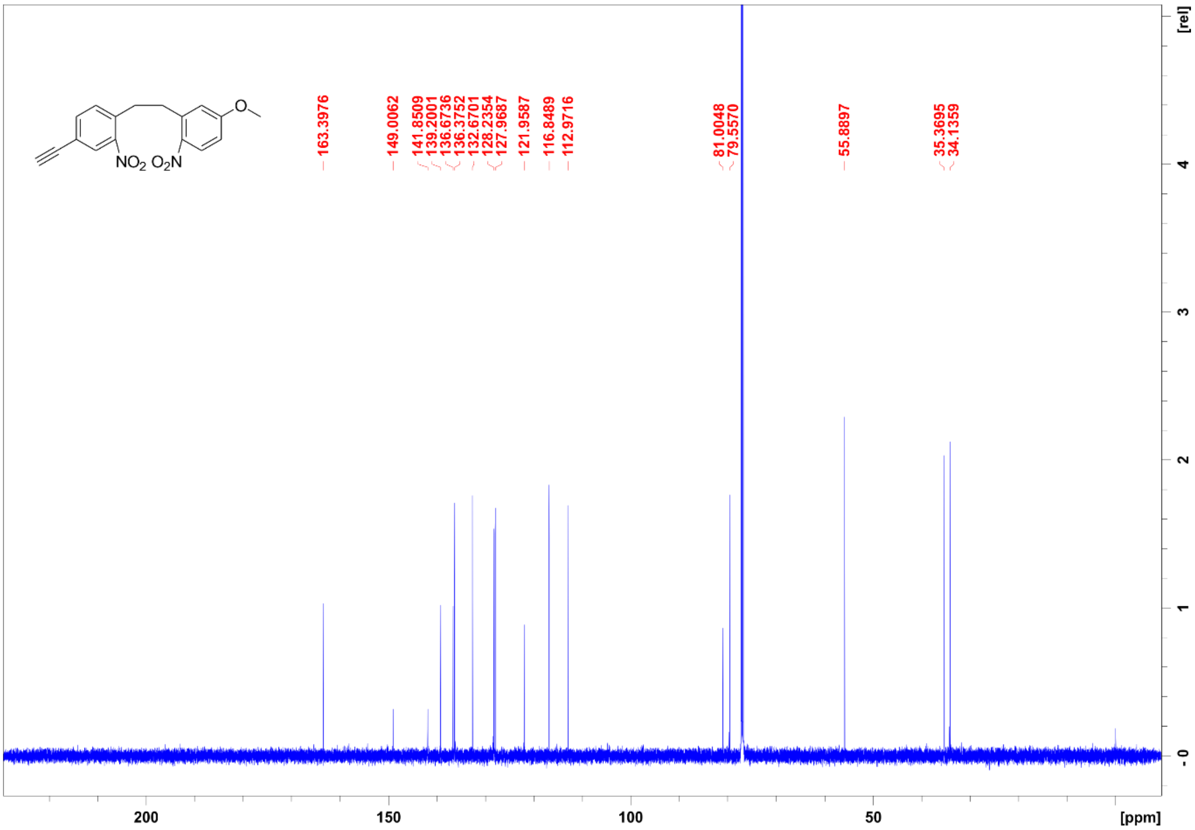

Figure S10. ^{13}C NMR spectrum (150.9 MHz, $CDCl_3$) of compound **12**.

III.6 (*Z*)-8-Ethynyl-2-methoxy-11,12-dihydrodibenzo[*c*,*g*][1,2]diazocine

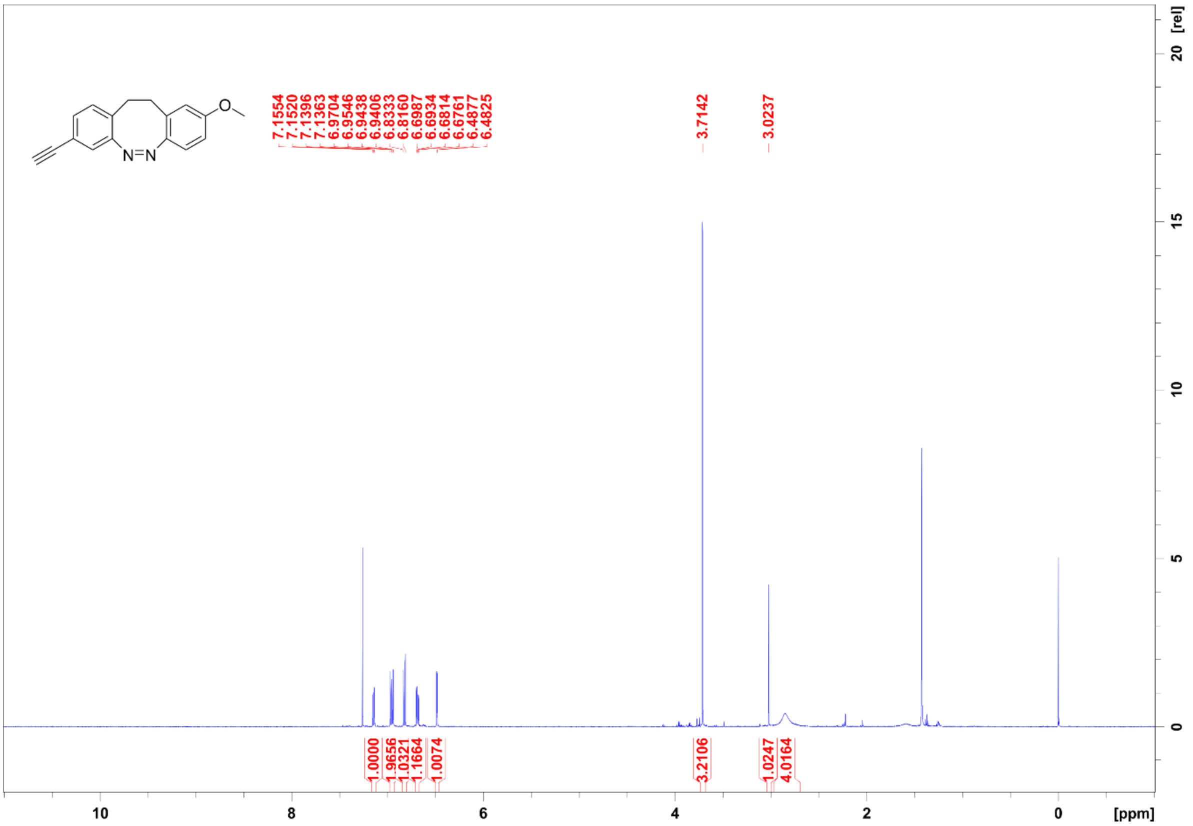

Figure S11. ^{1}H NMR spectrum (500.1 MHz, $CDCl_3$) of compound **13**.

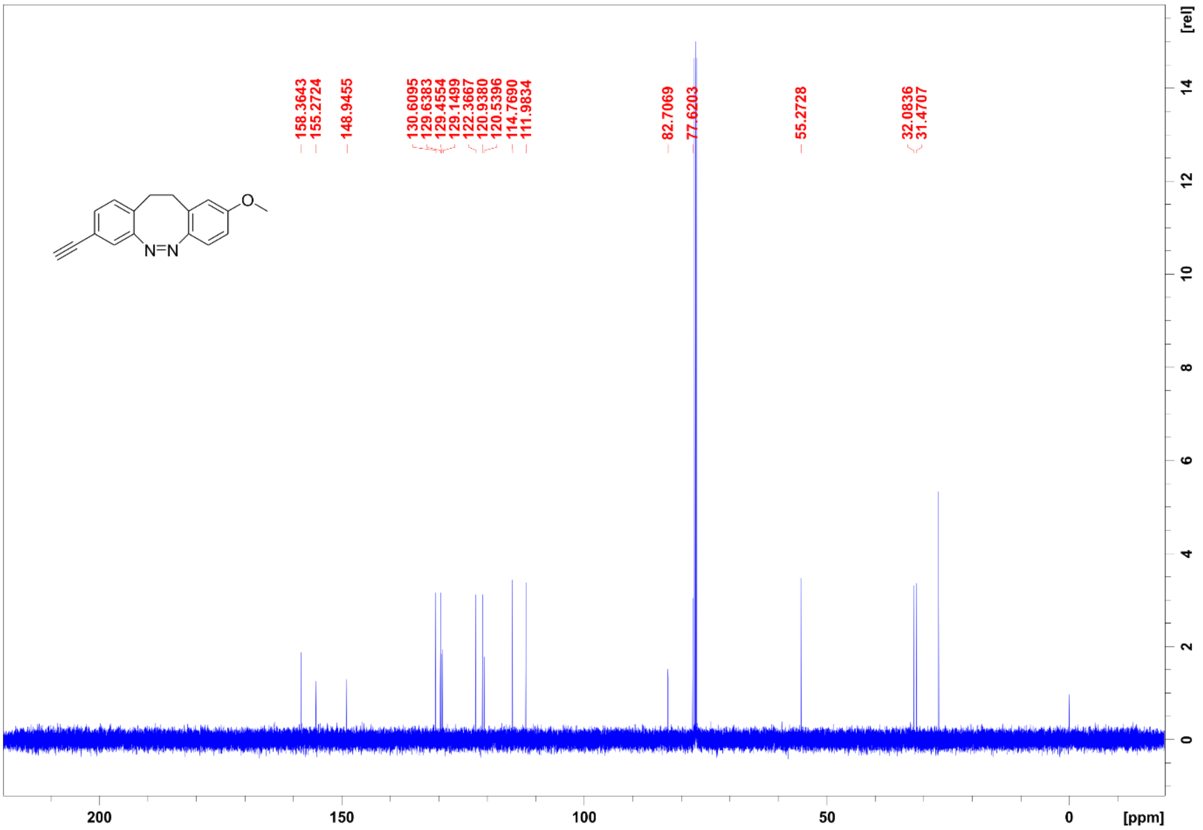

Figure S12. ^{13}C NMR spectrum (125.8 MHz, $CDCl_3$) of compound **13**.

III.7 (*Z*)-12c-(9-Methoxy-11,12-dihydrodibenzo[c,g][1,2]diazocin-3-yl)ethynyl-4,8,12-tri-*n*-octyl-4,8,12-triazatriangulene

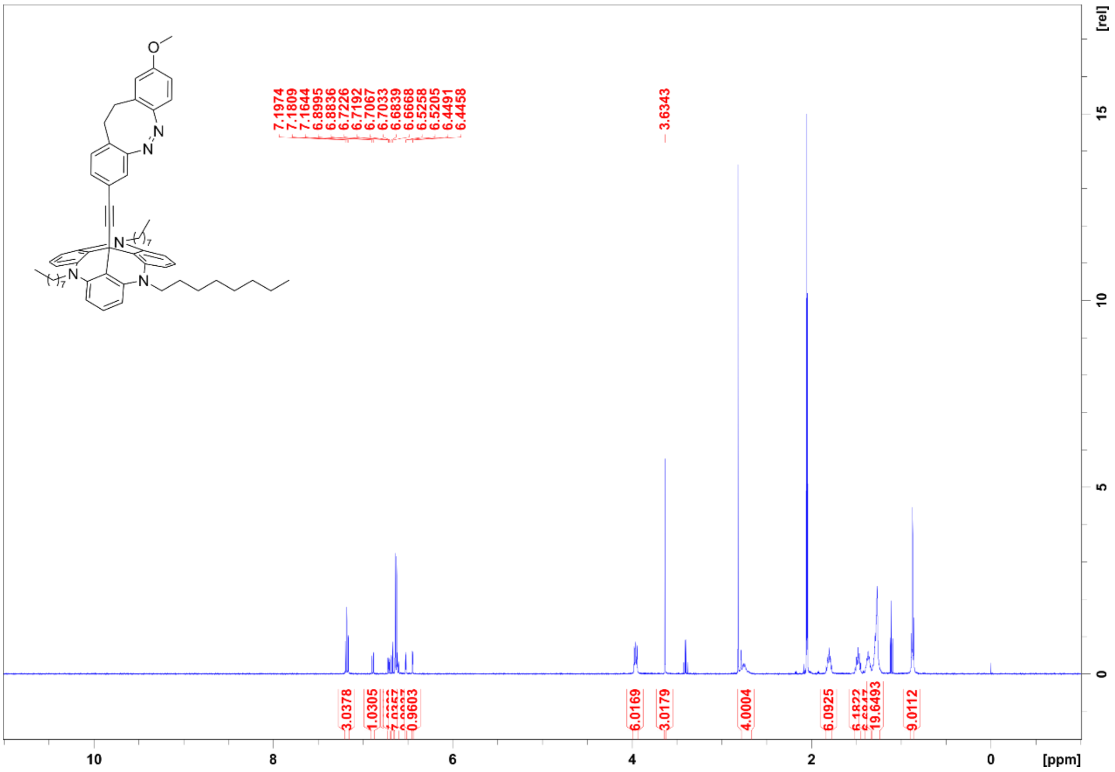

Figure S13. ^{1}H NMR spectrum (500.1 MHz, acetone-d$_6$) of compound **2**.

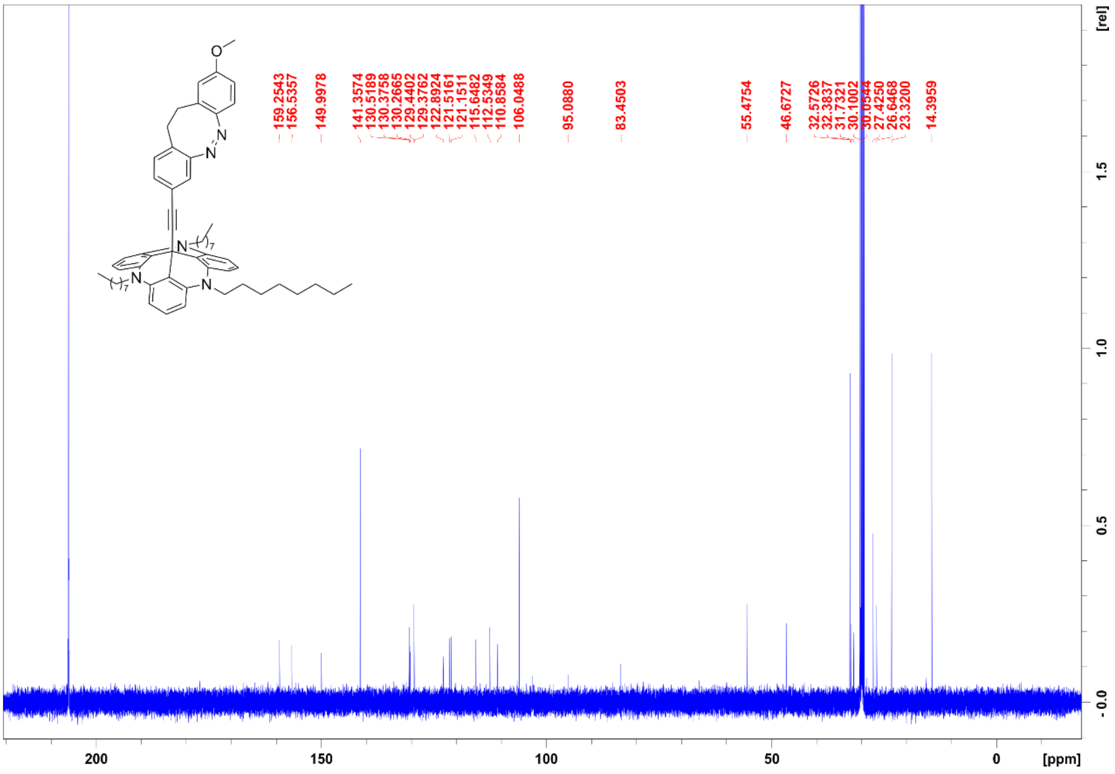

Figure S14. ^{13}C NMR spectrum (125.8 MHz, acetone-d$_6$) of compound **2**.

IV. UV–vis absorption spectra

IV.1 Methods

UV-Vis spectra were recorded on a PerkinElmer Lambda 650 Photospectrometer in a 1 cm path length quartz cuvette. Irradiation of UV–vis samples were carried out at 25 °C using a custom-made LED positioned at a distance of 1 cm from the sample.

IV.2 UV–vis spectra

Compound 1:

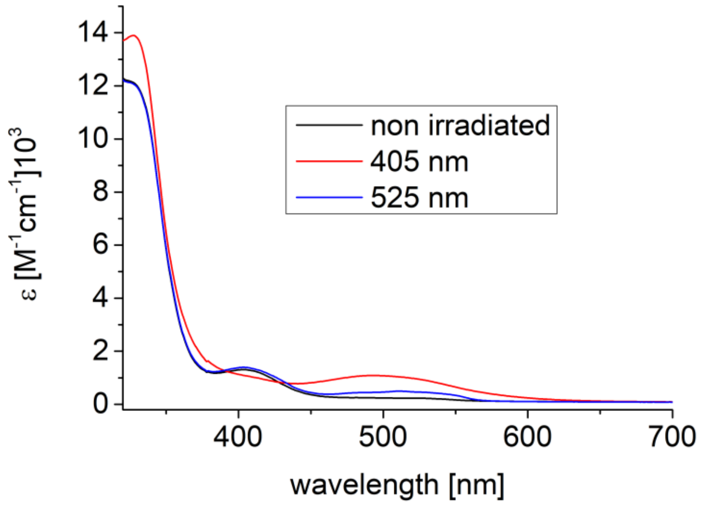

Figure S15. UV–vis spectra of compound **1** in tetrahydrofuran at room temperature (68 μmol/L). Upon irradiation with 405 nm and 525 nm.

Compound 2:

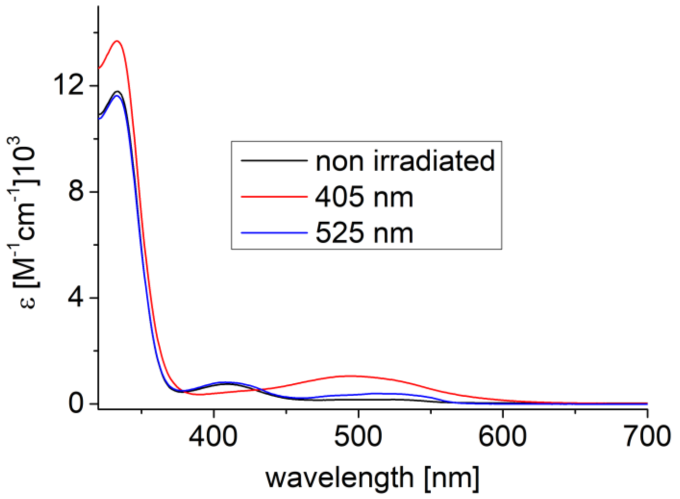

Figure S16. UV–vis spectra of compound **2** in tetrahydrofuran at room temperature (68 μmol/L). Upon irradiation with 405 nm and 525 nm.

V. Kinetic studies in solution by ^{1}H NMR spectroscopy

V.1 Thermal isomerization measurements by ^{1}H NMR

V.1.1 Compound 1: (*Z*)-12c-(8-methoxy-11,12-dihydrodibenzo[c,g][1,2]diazocin-2-yl)ethynyl-4,8,12-tri-*n*-octyl-4,8,12-triazatriangulene

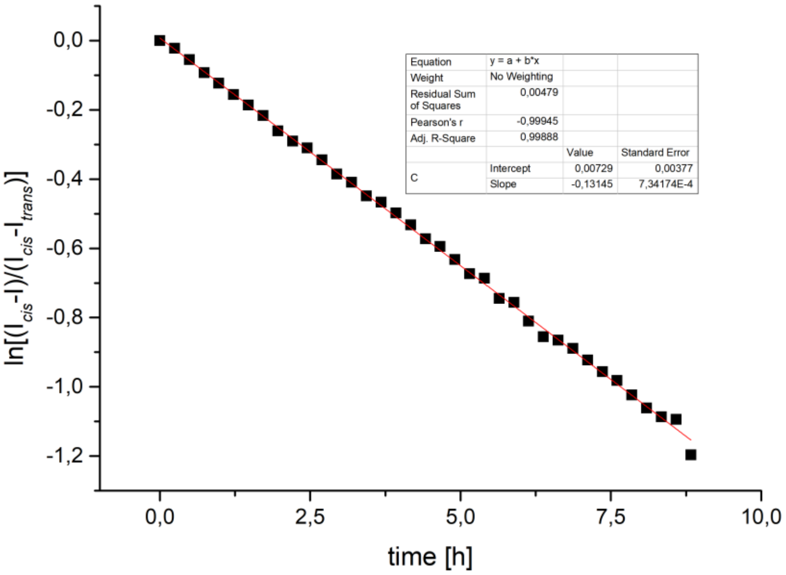

Figure S17. Determination of the thermal isomerization rate *k* of **1b→1a** by ^{1}H NMR spectroscopy (deuterated toluene, 290.5 K, 2.05 mmol/L). (I_{cis}-I): ^{1}H NMR integral of the CH_3 signal of the methoxy group at time *t*, (I_{cis}-I_{trans}) corresponding ^{1}H integral at *t* = 0. A rate constant of $k = 0.131$ [s^{-1}] was determined from a linear fit of the ln[(I_{cis}-I)/(I_{cis}-I_{trans})]/*t* curve. The half-life of **1** at 290.5 K in toluene was determined as 5.27 h.

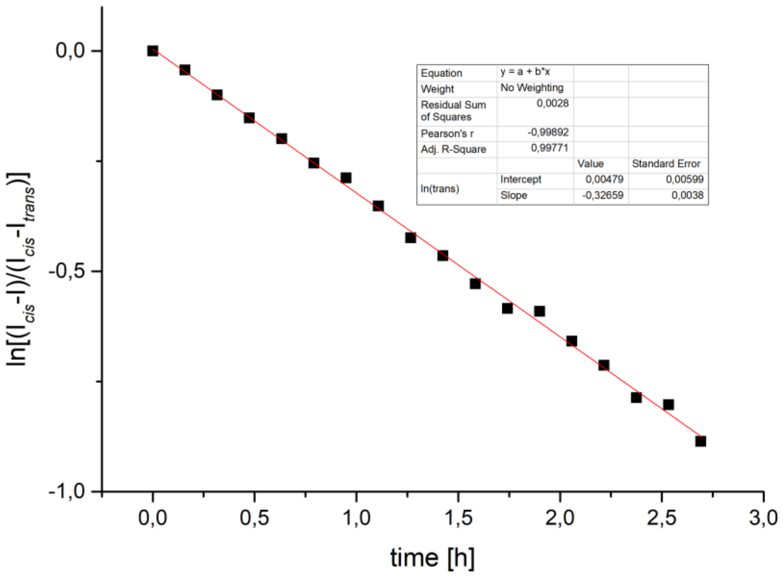

Figure S18. Determination of the thermal isomerization rate k of **1b**→**1a** by ^{1}H NMR spectroscopy (deuterated toluene, 298 K, 2.05 mmol/L). (I_{cis}-I): ^{1}H NMR integral of the CH_3 signal of the methoxy group at time *t*, (I_{cis}-I_{trans}) corresponding ^{1}H integral at t = 0. A rate constant of k = 0.327 [s^{-1}] was determined from a linear fit of the ln[(I_{cis}-I)/(I_{cis}-I_{trans})]/*t* curve. The half-life of **1** at 298 K in toluene was determined as 2.12 h.

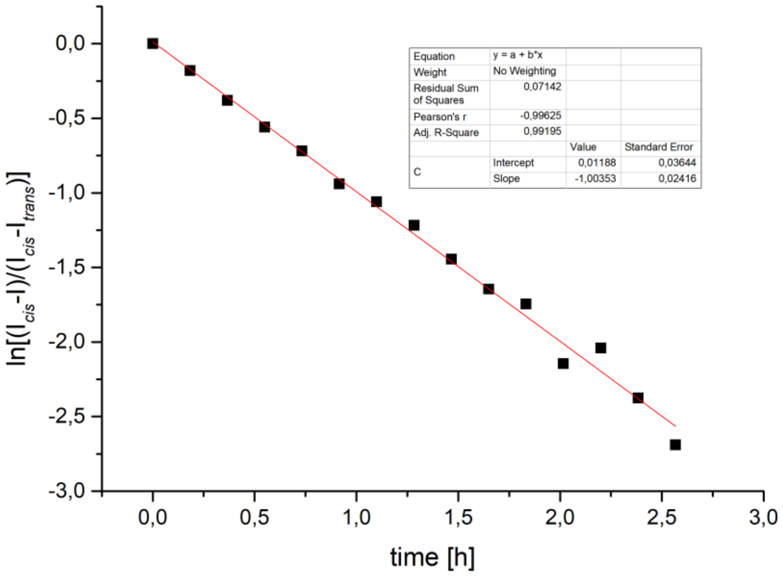

Figure S19. Determination of the thermal isomerization rate *k* of **1b**→**1a** by ^{1}H NMR spectroscopy (deuterated toluene, 308 K, 2.05 mmol/L). (I_{cis}-I): ^{1}H NMR integral of the CH_3 signal of the methoxy group at time *t*, (I_{cis}-I_{trans}) corresponding ^{1}H integral at t = 0. A rate constant of k = 1.00 [s^{-1}] was determined from a linear fit of the ln[(I_{cis}-I)/(I_{cis}-I_{trans})]/*t* curve. The half-life of **1** at 308 K in toluene was determined as 0.69 h.

V.1.2 Compound 2: (*Z*)-12c-(9-methoxy-11,12-dihydrodibenzo(c,g)(1,2)diazocin-3-yl)ethynyl-4,8,12-tri-*n*-octyl-4,8,12-triazatriangulene

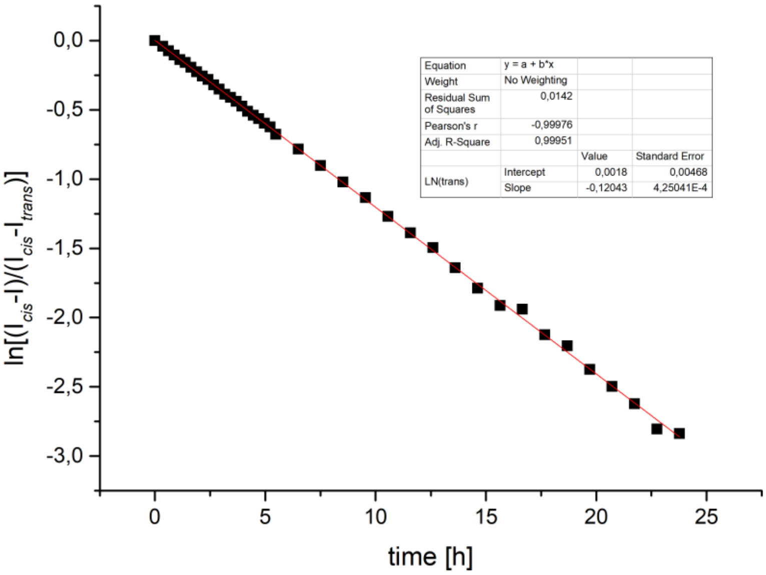

Figure S20. Determination of the thermal isomerization rate *k* of **2b→2a** by ^{1}H NMR spectroscopy (deuterated toluene, 290.5 K, 2.27 mmol/L). (I_{cis}-I): ^{1}H NMR integral of the CH_3 signal of the methoxy group at time *t*, (I_{cis}-I_{trans}) corresponding ^{1}H integral at t = 0. A rate constant of k = 0.120 [s^{-1}] was determined from a linear fit of the ln[(I_{cis}-I)/(I_{cis}-I_{trans})]/*t* curve. The half-life of **2** at 290.5 K in toluene was determined as 5.76 h.

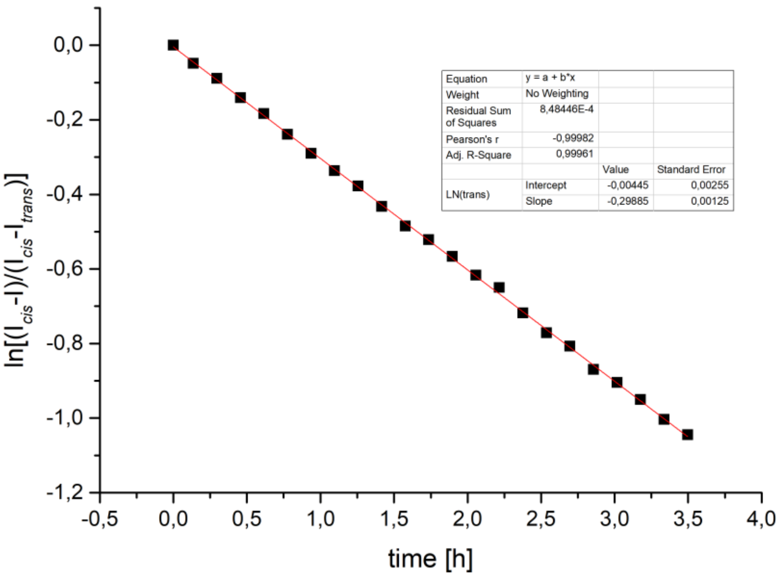

Figure S21. Determination of the thermal isomerization rate *k* of **2b**→**2a** by ^{1}H NMR spectroscopy (deuterated toluene, 298 K, 2.27 mmol/L). (I_{cis}-I): ^{1}H NMR integral of the CH_3 signal of the methoxy group at time *t*, (I_{cis}-I_{trans}) corresponding ^{1}H integral at t = 0. A rate constant of k = 0.299 [s^{-1}] was determined from a linear fit of the ln[(I_{cis}-I)/(I_{cis}-I_{trans})]/*t* curve. The half-life of **2** at 298 K in toluene was determined as 2.32 h.

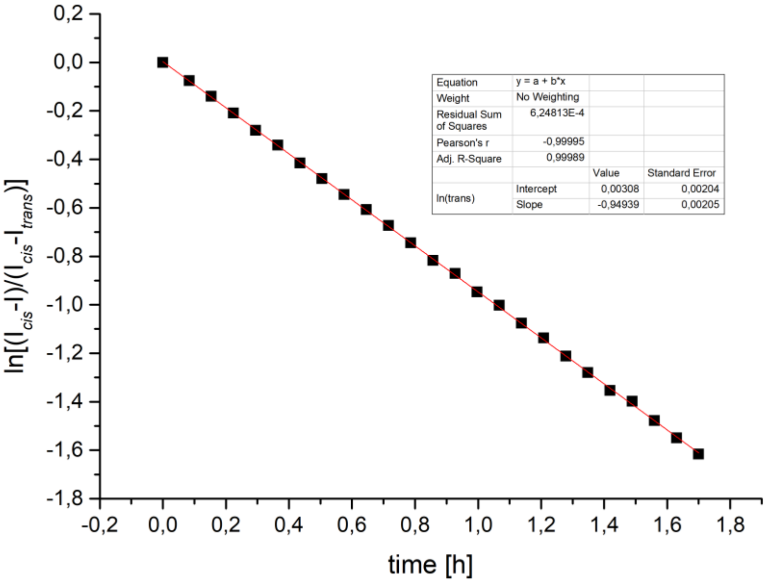

Figure S22. Determination of the thermal isomerization rate *k* of **2b**→**2a** by ^{1}H NMR spectroscopy (deuterated toluene, 308 K, 2.27 mmol/L). (I_{cis}-I): ^{1}H NMR integral of the CH_3 signal of the methoxy group at time *t*, (I_{cis}-I_{trans}) corresponding ^{1}H integral at t = 0. A rate constant of k = 0.949 [s^{-1}] was determined from a linear fit of the ln[(I_{cis}-I)/(I_{cis}-I_{trans})]/*t* curve. The half-life of **2** at 308 K in toluene was determined as 0.73 h.

V.2 Arrhenius Plots in solution

V.2.1 Compound 1: (*Z*)-12c-(8-methoxy-11,12-dihydrodibenzo[c,g][1,2]diazocin-2-yl)ethynyl-4,8,12-tri-*n*-octyl-4,8,12-triazatriangulene

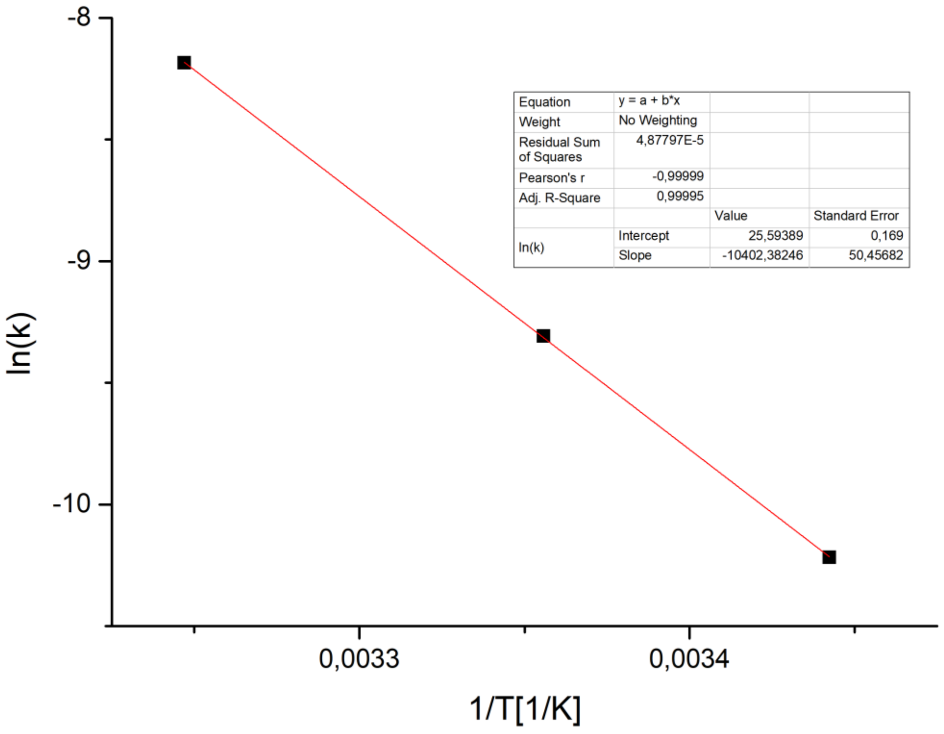

Figure S23. Arrhenius plot of the thermal isomerization of diazocine-TATA **1** in toluene-d_8 (2.05 mmol/L). An activation energy of 86.5 kJ/mol was determined from the linear fit.

V.2.2 Compound 2: (*Z*)-12c-(9-methoxy-11,12-dihydrodibenzo[c,g][1,2]diazocin-3-yl)ethynyl-4,8,12-tri-*n*-octyl-4,8,12-triazatriangulene

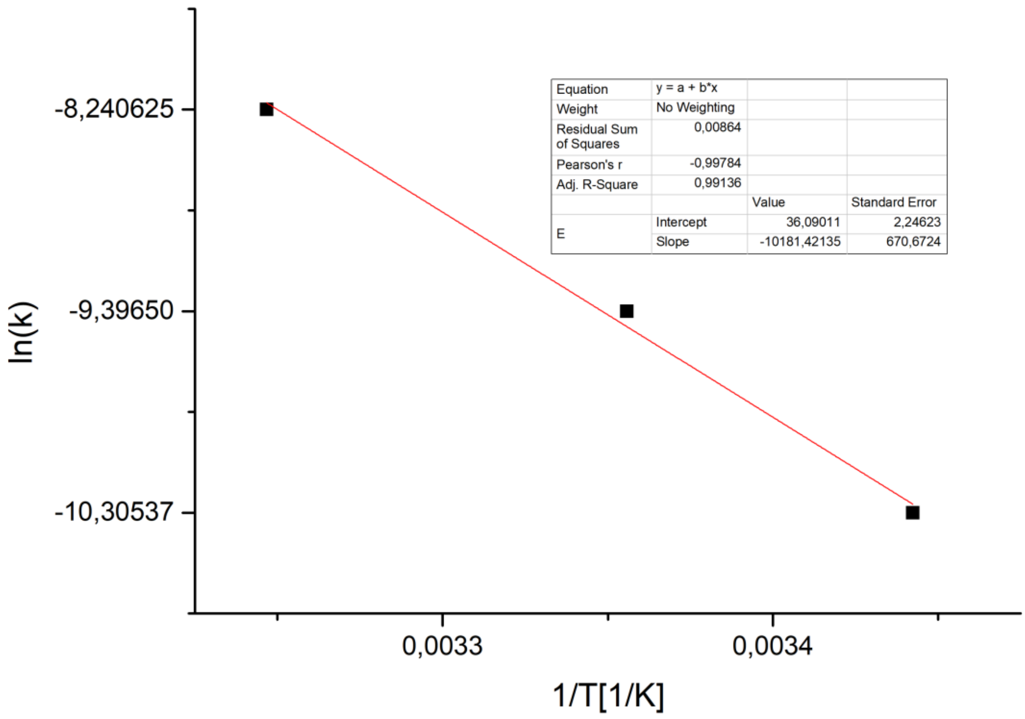

Figure S24. Arrhenius plot of the thermal isomerization of diazocine-TATA **1** in toluene-d_8 (2.27 mmol/L). An activation energy of 84.7 kJ/mol was determined from the linear fit.

VI. Calculations

The geometry optimizations were carried out using density functional theory with the Minnesota functional M06-2X [4] including Grimmes D3 [5] dispersion correction and the large triple zeta basis def2-TZVP [6]. This theoretical level performed well in Grimme's latest study on basic properties of a large set of organic molecules [7]. The calculations were performed with Turbomole7.2 [8], including the m4 grid (in Turbomole nomenclature) and resolution-of-identity (RI) with multipole accelerated RI-J (marij) to speed up the calculations. All stationary points are characterized by frequency calculations.

Coordinates

1a *cis para*-diazocine platform molecule

$E_{M062X\text{-}D3/def2TZVP}$ = -1735.7479869

Nimag = 0

C	0.8423706	1.1888220	-0.6747150
C	-0.1996660	2.0068443	-1.1074483
C	-0.1398925	3.3967441	-1.0422542
C	1.0223796	3.9601501	-0.5091298
C	2.0852794	3.1594786	-0.1005557
C	1.9973550	1.7838344	-0.1632224
H	-1.0914091	1.5383759	-1.5056817
H	2.9819589	3.6400909	0.2693096
C	-1.3105728	4.1869891	-1.5808886
C	-1.7461674	5.4307277	-0.7760201
H	-1.0924538	4.4946436	-2.6076251
H	-2.1542639	3.4983177	-1.6405533
C	-1.5420640	5.2802242	0.7041029
H	-1.1986834	6.3052325	-1.1276249
H	-2.8003327	5.6183047	-0.9827574
C	-0.2844198	5.5415248	1.2574631
N	0.7413389	6.0835543	0.4087727
N	1.2900393	5.3763382	-0.4349021
C	-2.5394135	4.8649177	1.5709273
C	-2.3125004	4.7020769	2.9341852
C	-1.0516817	4.9786282	3.4537246
C	-0.0360076	5.4225193	2.6090289
H	0.9345117	5.6637019	3.0215445
H	-3.5272321	4.6583588	1.1749579
C	3.9125498	-4.1448213	0.3982673
C	4.6345339	-4.2440201	-0.7810573
C	4.0471275	-4.0005035	-2.0127256
C	2.7057736	-3.6212184	-2.0624485
C	1.9807273	-3.4750703	-0.8830149
C	2.5715400	-3.7649591	0.3435754
H	4.3769412	-4.3609439	1.3520549
H	5.6758958	-4.5362731	-0.7404083
H	4.6160706	-4.1032279	-2.9281033
C	0.5801611	-2.9076635	-0.9274357
C	-0.0865050	-3.2926499	-2.2284278
C	-0.2242457	-3.4401278	0.2368146
C	0.6802168	-3.4448358	-3.3805660
C	0.0558928	-3.6574386	-4.6095389
C	-1.3272498	-3.7326336	-4.6601775
C	-2.1007592	-3.6268692	-3.5146184
C	-1.4719191	-3.4135487	-2.2880545
H	0.6499490	-3.7641717	-5.5083383
H	-1.8132084	-3.8967404	-5.6133066
H	-3.1789158	-3.7116775	-3.5641797
C	-1.6068547	-3.5584966	0.1271758
C	-2.3664292	-3.9129133	1.2420571
C	-1.7241891	-4.1607832	2.4452278
C	-0.3444466	-4.0889102	2.5578823
C	0.4109032	-3.7338574	1.4403335
H	-3.4428446	-3.9956847	1.1611128
N	1.7986504	-3.6621140	1.4954364
H	2.2277130	-3.9774921	2.3498833
N	2.0648118	-3.3781377	-3.2723777
H	2.5885516	-3.5904101	-4.1056892
N	-2.2006654	-3.3137539	-1.1070004
H	-3.1844787	-3.5174147	-1.1741062
H	-2.3115962	-4.4356067	3.3117727
H	0.1467189	-4.3085544	3.4973437
H	-3.1193570	4.3721736	3.5712556
H	2.8166211	1.1625062	0.1714491
C	0.7345624	-0.2343858	-0.7608235
C	0.6573516	-1.4322857	-0.8353981
O	-0.7193084	4.8615394	4.7621776
C	-1.7112975	4.4038811	5.6523843
H	-2.5641030	5.0876124	5.6803824
H	-1.2455211	4.3680744	6.6333135
H	-2.0578418	3.4040073	5.3772583

1b *trans-twist para*-diazocine platform molecule

$E_{M062x\text{-}D3/def2TZVP}$ = -1735.735229021

Nimag = 0

C	0.8401254	0.1948635	0.0360794
C	-0.2729335	1.0400154	-0.0243101
C	-0.1386510	2.4177595	-0.1184809
C	1.1692801	2.9299883	-0.1589074
C	2.2860807	2.1127853	-0.1424469
C	2.1232416	0.7436426	-0.0203797
H	-1.2612185	0.5966497	-0.0038849
H	3.2718125	2.5533381	-0.2131845
C	-1.3762462	3.3088971	-0.2078395
C	-1.3958483	4.4404894	-1.2796657
H	-1.5828978	3.7407077	0.7731500
H	-2.2121142	2.6432411	-0.4262808
C	-1.0454856	5.8625000	-0.8489445
H	-2.4099923	4.4858328	-1.6793384
H	-0.7578972	4.1453934	-2.1157176
C	0.0446053	6.1544622	-0.0072817
N	0.6676551	4.9751295	0.4861435
N	1.2069869	4.3266472	-0.4148385
C	-1.8014483	6.9456693	-1.2654587
C	-1.4995793	8.2533712	-0.8897991
C	-0.4193272	8.4956810	-0.0487556
C	0.3481191	7.4278275	0.4164893
H	1.1741032	7.6128466	1.0894853
H	-2.6574324	6.7748141	-1.9084151
C	3.5638146	-5.2305087	1.7091597
C	4.2663082	-5.5114055	0.5476077
C	3.6905641	-5.3535140	-0.7035748
C	2.3831539	-4.8759661	-0.7939827
C	1.6815291	-4.5471477	0.3626292
C	2.2567143	-4.7528786	1.6135244
H	4.0166120	-5.3816756	2.6808333
H	5.2813791	-5.8800634	0.6200090
H	4.2410387	-5.6009943	-1.6023805
C	0.3289959	-3.8804263	0.2572901
C	-0.3762696	-4.3491317	-0.9954210
C	-0.5054668	-4.2208910	1.4711478
C	0.3668245	-4.6830394	-2.1244616
C	-0.2819383	-4.9747604	-3.3241070
C	-1.6670613	-4.9435670	-3.3672287
C	-2.4206648	-4.6546030	-2.2400173
C	-1.7673524	-4.3637036	-1.0426123
H	0.2940887	-5.2256625	-4.2058178
H	-2.1720674	-5.1679745	-4.2978623
H	-3.5024669	-4.6564358	-2.2814871
C	-1.8939926	-4.2381829	1.3740998
C	-2.6704482	-4.4080346	2.5200733
C	-2.0402677	-4.5761731	3.7433375
C	-0.6584129	-4.6055172	3.8483196
C	0.1139022	-4.4345541	2.6994767
H	-3.7506721	-4.4106999	2.4483676
N	1.5031637	-4.4697177	2.7472178
H	1.9131286	-4.7240231	3.6308465
N	1.7533543	-4.7140140	-2.0231881
H	2.2519580	-5.0547658	-2.8288416
N	-2.4760349	-4.0792681	0.1203924
H	-3.4736538	-4.2078232	0.0747186
H	-2.6411549	-4.7051841	4.6343589
H	-0.1789633	-4.7616051	4.8063518
H	-2.1184867	9.0621580	-1.2483986
H	2.9820643	0.0874583	0.0154085
C	0.6625517	-1.2214745	0.1247796
C	0.5137014	-2.4133541	0.1878260
O	-0.0431878	9.7250870	0.3812710
C	-0.7941606	10.8319233	-0.0627180
H	-0.7673156	10.9161225	-1.1524606
H	-0.3318585	11.7103496	0.3792683

H -1.8338887 10.7629892 0.2688271

1c *trans-chair para*-diazocine platform molecule

$E_{M062x\text{-}D3/def2TZVP}$ = -1735.731545735

Nimag = 0

C 0.7082396 0.2261391 0.3888250
C -0.4046217 1.0614675 0.2314074
C -0.2849941 2.4414148 0.2850565
C 1.0007728 2.9625528 0.4950667
C 2.1123435 2.1672652 0.6774918
C 1.9642902 0.7891610 0.6158220
H -1.3765155 0.6102457 0.0722836
H 3.0803611 2.6185564 0.8488864
C -1.5061101 3.3514154 0.2289882
C -1.6607090 4.3745881 -0.9415195
H -1.5656470 3.8990432 1.1732130
H -2.3764585 2.6950228 0.1951461
C -1.1663037 5.8043388 -0.7656366
H -2.7274781 4.4439738 -1.1594537
H -1.1907471 3.9507376 -1.8329601
C 0.2023232 6.0942363 -0.6210724
N 0.9487146 4.8766153 -0.5838408
N 0.9462892 4.3884030 0.5463486
C -2.0148260 6.8953695 -0.8368125
C -1.5428375 8.2069148 -0.7754406
C -0.1791626 8.4431141 -0.6536034
C 0.7104348 7.3675658 -0.5800397
H 1.7703948 7.5530838 -0.4742390
H -3.0801986 6.7293631 -0.9492613
C 3.5743751 -5.1778347 1.6618947
C 4.3126377 -5.3407285 0.4998894
C 3.7562225 -5.1153072 -0.7495741
C 2.4308280 -4.6891601 -0.8360845
C 1.6909782 -4.4801618 0.3245306
C 2.2491018 -4.7524653 1.5704019
H 4.0140529 -5.3795116 2.6304364
H 5.3414802 -5.6696257 0.5691698
H 4.3367669 -5.2682985 -1.6506197
C 0.3127289 -3.8653812 0.2367203
C -0.3442793 -4.2665333 -1.0642772
C -0.5305801 -4.3325596 1.4012316
C 0.4365903 -4.4806247 -2.1968641
C -0.1730496 -4.7078390 -3.4304774
C -1.5569448 -4.7354486 -3.5047030
C -2.3461503 -4.5684054 -2.3773795
C -1.7319121 -4.3403067 -1.1460741
H 0.4325221 -4.8623801 -4.3146005
H -2.0316730 -4.9100628 -4.4615777
H -3.4256106 -4.6166721 -2.4445513
C -1.9144591 -4.4048704 1.2698584
C -2.7057063 -4.6940063 2.3816618
C -2.0940015 -4.9238661 3.6041398
C -0.7147348 -4.8982802 3.7399250
C 0.0722537 -4.6091744 2.6252323
H -3.7829147 -4.7398869 2.2837639
N 1.4605433 -4.5859946 2.7037064
H 1.8630241 -4.8885180 3.5755163
N 1.8207175 -4.4604832 -2.0646164
H 2.3506686 -4.7175443 -2.8812164
N -2.4773722 -4.1802558 0.0176199
H -3.4666843 -4.3500615 -0.0617535
H -2.7062303 -5.1471152 4.4684313
H -0.2482327 -5.1022131 4.6953042
H -2.2460488 9.0242121 -0.8319690
H 2.8198825 0.1391608 0.7391279
C 0.5573863 -1.1953463 0.3306599
C 0.4404436 -2.3915078 0.2864734
O 0.3839256 9.6744167 -0.5924437
C -0.4759518 10.7901697 -0.6398201
H -1.0379749 10.8166874 -1.5770790
H 0.1608989 11.6680495 -0.5756017
H -1.1750720 10.7851469 0.2009318

1 TS *twist2cis para*-diazocine platform molecule

$E_{M062x\text{-}D3/def2TZVP}$ = -1735.699182956

Nimag = 1 (XXX)

C 0.3680808 3.0635202 -1.7967020
H 1.4206581 3.3321159 -1.9122578
H 0.1870829 2.2465766 -2.4948836
C 1.5150269 7.2471821 -3.6040155
C 0.5711058 6.2222035 -3.5199735
C 0.3483589 5.5219119 -2.3548531
C 1.0645587 5.9255925 -1.1951874
C 2.0245940 6.9382330 -1.2563942
C 2.2557916 7.5756926 -2.4706701
C 0.1568622 2.5000834 -0.4078982
N 0.7453893 5.2639332 -0.0846180
N 0.1954972 4.6962046 0.8387140
C 0.1274746 3.2532519 0.7772774
C -0.0218188 1.1277480 -0.2776612
C -0.2307323 1.2843465 2.1192757
C -0.0876462 2.6528749 2.0121935
H 1.6748239 7.7496302 -4.5452462
H 0.0278154 5.9351164 -4.4133732
H 2.5754089 7.2395767 -0.3776367
H -0.0246564 0.5086138 -1.1662823
H -0.3831785 0.8111169 3.0794798
H -0.1375282 3.2948706 2.8825064
C -0.4954302 4.2871743 -2.2331561
H -0.9593991 4.0757152 -3.1961138
H -1.3122196 4.4290282 -1.5196740
O 3.2142652 8.5378853 -2.4446310
C 3.4409836 9.2685310 -3.6271398
H 4.2160590 9.9932868 -3.3929059
H 3.7849098 8.6185118 -4.4362434
H 2.5370770 9.7939634 -3.9469266
C -0.2002532 0.5049472 0.9612639
C 2.4895026 -4.8998917 2.7418355
C 3.2222827 -5.2009076 1.6042425
C 2.6809430 -5.0620322 0.3356202
C 1.3783239 -4.5821830 0.2022019
C 0.6465310 -4.2344514 1.3341981
C 1.1870723 -4.4209895 2.6034216
H 2.9158329 -5.0356485 3.7276160
H 4.2340577 -5.5703028 1.7098117
H 3.2553655 -5.3243057 -0.5437306
C -0.7029324 -3.5698917 1.1822805
C -1.3739700 -4.0543798 -0.0824149
C -1.5699436 -3.8903029 2.3782215
C -0.6008122 -4.4047363 -1.1858380
C -1.2174065 -4.7100340 -2.3990481
C -2.6008789 -4.6739611 -2.4807512
C -3.3844571 -4.3688074 -1.3786551
C -2.7635184 -4.0658609 -0.1670248
H -0.6182965 -4.9748522 -3.2610530
H -3.0810260 -4.9079900 -3.4220505
H -4.4646521 -4.3669946 -1.4502161
C -2.9554900 -3.9053726 2.2436902
C -3.7626836 -4.0525310 3.3715127
C -3.1657951 -4.2001418 4.6138920
C -1.7871955 -4.2313172 4.7567481
C -0.9839561 -4.0828977 3.6262269
H -4.8406042 -4.0527639 3.2711848
N 0.4038289 -4.1172850 3.7118856
H 0.7889869 -4.3607933 4.6097254
N 0.7827099 -4.4360360 -1.0463831
H 1.3015911 -4.7930127 -1.8320117
N -3.5022211 -3.7674134 0.9725318
H -4.4998958 -3.8822935 0.9006233
H -3.7906782 -4.3115957 5.4905843
H -1.3342079 -4.3717034 5.7299641
C -0.3696216 -0.9118524 1.0349016
C -0.5157470 -2.1039058 1.0999901

2a *cis meta*-diazocine platform molecule

$E_{M062x\text{-}D3/def2TZVP}$ = -1735.747650270

Nimag = 0

C 0.1005678 2.0463851 -1.6611740
C 0.1244073 3.4153545 -1.4781007
C 0.7130306 4.0163742 -0.3630355
C 1.2868067 3.1637902 0.5831543
C 1.2988344 1.7871428 0.3978979
C 0.6918946 1.2092155 -0.7130393
H -0.3378961 4.0541600 -2.2213399
H 1.7871697 1.1619962 1.1338705
C 0.7099925 5.5242194 -0.2786540
C 0.4815875 6.1424476 1.1150637
H 1.6510426 5.9104667 -0.6810430
H -0.0769943 5.8768182 -0.9470539
C -0.4742917 5.3447437 1.9565171
H 1.4327406 6.2320394 1.6393818
H 0.0967099 7.1536497 0.9821977
C 0.0059671 4.2661577 2.7064472
N 1.4213897 4.0190734 2.7520312
N 2.0108712 3.5881256 1.7613586
C -1.8264152 5.6321308 2.0079822
C -2.6969736 4.8639713 2.7811628
C -2.2029403 3.8069115 3.5385827
C -0.8410511 3.5297002 3.5119781
H -0.4364720 2.7266271 4.1144295
H -2.2356606 6.4635855 1.4468042
C 4.0897990 -3.9732270 0.1421640
C 4.8082534 -4.0066847 -1.0429093
C 4.2012367 -3.7644020 -2.2653270
C 2.8420994 -3.4536913 -2.2988122
C 2.1181236 -3.3737001 -1.1125317
C 2.7309982 -3.6613529 0.1036630
H 4.5710484 -4.1868645 1.0881025
H 5.8635872 -4.2451046 -1.0148008
H 4.7690474 -3.8147211 -3.1856659
C 0.6889933 -2.8809961 -1.1359764
C 0.0353231 -3.2679406 -2.4425163
C -0.0786814 -3.4833737 0.0191137
C 0.8015296 -3.3523428 -3.6018550
C 0.1811351 -3.5685847 -4.8323503
C -1.1965427 -3.7149123 -4.8774026
C -1.9670395 -3.6775307 -3.7255399
C -1.3422967 -3.4604759 -2.4974708
H 0.7740172 -3.6230725 -5.7365064
H -1.6794983 -3.8821422 -5.8315473
H -3.0395489 -3.8177913 -3.7713730
C -1.4543099 -3.6684836 -0.0855904
C -2.1878228 -4.0825863 1.0260646
C -1.5260397 -4.3208304 2.2205889
C -0.1507140 -4.1830826 2.3270657
C 0.5786095 -3.7681373 1.2128009
H -3.2594800 -4.2162373 0.9499809
N 1.9610137 -3.6247413 1.2618042

H	2.4118516	-3.9345560	2.1070827
N	2.1813889	-3.2148393	-3.4994084
H	2.7099417	-3.3819104	-4.3399593
N	-2.0670041	-3.4282827	-1.3108427
H	-3.0412989	-3.6733052	-1.3771396
H	-2.0935535	-4.6405729	3.0848896
H	0.3566416	-4.3956912	3.2595875
C	0.6842865	-1.4072352	-0.9999765
C	0.6860142	-0.2113059	-0.8745509
H	-0.3703190	1.6143836	-2.5337616
H	-2.8539400	3.2060591	4.1558922
O	-4.0021655	5.2313538	2.7416285
C	-4.9167402	4.4812763	3.5077115
H	-5.8942076	4.9215863	3.3313237
H	-4.6799219	4.5375812	4.5736596
H	-4.9281323	3.4333820	3.1952714

2b *trans-twist meta*-diazocine platform molecule

$E_{M062x\text{-}D3/def2TZVP}$ = -1735.735003400

Nimag = 0

C	-2.1201189	1.1640095	0.3349273
C	-2.0441739	2.5472550	0.4061257
C	-0.8270935	3.2145272	0.3230141
C	0.3189743	2.4198439	0.1554423
C	0.2658510	1.0463880	0.0392032
C	-0.9668891	0.3987491	0.1512976
H	-3.0763636	0.6637760	0.4063100
H	-2.9545174	3.1229730	0.5277103
H	1.1706741	0.4763477	-0.1233989
C	-0.7594651	4.7373026	0.3952480
C	0.1419966	5.4914029	-0.6287705
H	-0.4746584	5.0371531	1.4064247
H	-1.7796641	5.0957765	0.2527965
C	1.5341116	5.9330840	-0.1804562
H	-0.3872137	6.4014109	-0.9138505
H	0.2279727	4.8943782	-1.5385119
C	2.3922304	5.1013830	0.5657239
N	1.7936363	3.8652328	0.9362226
N	1.4842046	3.2043174	-0.0606603
C	1.9837288	7.2076388	-0.4741841
C	3.2415142	7.6509661	-0.0576418
C	4.0608894	6.8208816	0.6990162
C	3.6115521	5.5463920	1.0289074
H	4.2233081	4.8903872	1.6343800
H	5.0296099	7.1500063	1.0441630
H	1.3632781	7.8987385	-1.0316748
O	3.5663912	8.9162750	-0.4230688
C	4.8167397	9.4175165	-0.0094065
H	4.8900513	9.4457824	1.0812618
H	4.8849324	10.4283219	-0.4021967
H	5.6368064	8.8160132	-0.4108599
C	-1.0401240	-1.0267811	0.0648989
C	-1.0894704	-2.2260766	-0.0088607
C	2.2092428	-4.8554828	1.2801515
C	2.9515644	-4.9384196	0.1123476
C	2.3773709	-4.7119779	-1.1288916
C	1.0278230	-4.3666047	-1.1999369
C	0.2812975	-4.2368301	-0.0321556
C	0.8599973	-4.5097456	1.2041016
H	2.6643420	-5.0572791	2.2415058
H	3.9993015	-5.2037807	0.1696615
H	2.9628532	-4.8018011	-2.0351253
C	-1.1313215	-3.7028733	-0.0990262
C	-1.7688725	-4.1052817	-1.4094306
C	-1.9409540	-4.2514505	1.0537321
C	-0.9812203	-4.2412175	-2.5492410
C	-1.5813166	-4.4709512	-3.7870490
C	-2.9614729	-4.5783564	-3.8587020
C	-3.7547439	-4.4885414	-2.7254234
C	-3.1500815	-4.2583607	-1.4899072
H	-0.9712587	-4.5650708	-4.6764685
H	-3.4288887	-4.7549405	-4.8188167
H	-4.8298392	-4.5975954	-2.7915149
C	-3.3186930	-4.4013471	0.9233912
C	-4.0870360	-4.7693676	2.0277829
C	-3.4577002	-4.9987404	3.2414473
C	-2.0817637	-4.8958517	3.3747525
C	-1.3176054	-4.5274066	2.2675762
H	-5.1600369	-4.8761676	1.9309700
N	0.0670082	-4.4247611	2.3431776
H	0.4907396	-4.7235177	3.2061270
N	0.3998080	-4.1403675	-2.4200155
H	0.9408966	-4.3425779	-3.2447035
N	-3.8988187	-4.1731661	-0.3203735
H	-4.8770293	-4.3974894	-0.4020633
H	-4.0520136	-5.2831363	4.1001247
H	-1.6002797	-5.1009146	4.3224631

2c *trans-chair meta*-diazocine platform molecule

$E_{M062x\text{-}D3/def2TZVP}$ = -1735.731545735

Nimag = 0

C	0.7082396	0.2261391	0.3888250
C	-0.4046217	1.0614675	0.2314074
C	-0.2849941	2.4414148	0.2850565
C	1.0007728	2.9625528	0.4950667
C	2.1123435	2.1672652	0.6774918
C	1.9642902	0.7891610	0.6158220
H	-1.3765155	0.6102457	0.0722836
H	3.0803611	2.6185564	0.8488864
C	-1.5061101	3.3514154	0.2289882
C	-1.6607090	4.3745881	-0.9415195
H	-1.5656470	3.8990432	1.1732130
H	-2.3764585	2.6950228	0.1951461
C	-1.1663037	5.8043388	-0.7656366
H	-2.7274781	4.4439738	-1.1594537
H	-1.1907471	3.9507376	-1.8329601
C	0.2023232	6.0942363	-0.6210724
N	0.9487146	4.8766153	-0.5838408
N	0.9462892	4.3884030	0.5463486
C	-2.0148260	6.8953695	-0.8368125
C	-1.5428375	8.2069148	-0.7754406
C	-0.1791626	8.4431141	-0.6536034
C	0.7104348	7.3675658	-0.5800397
H	1.7703948	7.5530838	-0.4742390
H	-3.0801986	6.7293631	-0.9492613
C	3.5743751	-5.1778347	1.6618947
C	4.3126377	-5.3407285	0.4998894
C	3.7562225	-5.1153072	-0.7495741
C	2.4308280	-4.6891601	-0.8360845
C	1.6909782	-4.4801618	0.3245306
C	2.2491018	-4.7524653	1.5704019
H	4.0140529	-5.3795116	2.6304364
H	5.3414802	-5.6696257	0.5691698
H	4.3367669	-5.2682985	-1.6506197
C	0.3127289	-3.8653812	0.2367203
C	-0.3442793	-4.2665333	-1.0642772
C	-0.5305801	-4.3325596	1.4012316
C	0.4365903	-4.4806247	-2.1968641
C	-0.1730496	-4.7078390	-3.4304774
C	-1.5569448	-4.7354486	-3.5047030
C	-2.3461503	-4.5684054	-2.3773795
C	-1.7319121	-4.3403067	-1.1460741
H	0.4325221	-4.8623801	-4.3146005
H	-2.0316730	-4.9100628	-4.4615777
H	-3.4256106	-4.6166721	-2.4445513
C	-1.9144591	-4.4048704	1.2698584
C	-2.7057063	-4.6940063	2.3816618
C	-2.0940015	-4.9238661	3.6041398
C	-0.7147348	-4.8982802	3.7399250
C	0.0722537	-4.6091744	2.6252323
H	-3.7829147	-4.7398869	2.2837639
N	1.4605433	-4.5859946	2.7037064
H	1.8630241	-4.8885180	3.5755163
N	1.8207175	-4.4604832	-2.0646164
H	2.3506686	-4.7175443	-2.8812164
N	-2.4773722	-4.1802558	0.0176199
H	-3.4666843	-4.3500615	-0.0617535
H	-2.7062303	-5.1471152	4.4684313
H	-0.2482327	-5.1022131	4.6953042
H	-2.2460488	9.0242121	-0.8319690
H	2.8198825	0.1391608	0.7391279
C	0.5573863	-1.1953463	0.3306599
C	0.4404436	-2.3915078	0.2864734
O	0.3839256	9.6744167	-0.5924437
C	-0.4759518	10.7901697	-0.6398201
H	-1.0379749	10.8166874	-1.5770790
H	0.1608989	11.6680495	-0.5756017
H	-1.1750720	10.7851469	0.2009318

TS *twist2cis meta*-diazocine platform molecule

$E_{M062x\text{-}D3/def2TZVP}$ = -1735.698417078

Nimag = 3 (-463.03; -18.65; -2.32 cm^{-1})

C	0.4799215	4.6077299	-1.0428513
H	1.5222966	4.8365489	-0.8076583
H	0.3519139	4.8427689	-2.0994513
C	1.4973969	8.1459795	1.8710647
C	0.5952935	7.6009605	0.9560476
C	0.3644579	6.2456689	0.8695926
C	1.0246275	5.4037812	1.8052373
C	1.9419284	5.9222488	2.7217206
C	2.1865545	7.2913146	2.7289713
C	0.2627984	3.1169252	-0.9037511
N	0.6974296	4.1155555	1.7230108
N	0.1460550	3.0388924	1.6188545
C	0.1601110	2.4172185	0.3088229
C	0.1568715	2.3517604	-2.0646549
C	-0.1240261	0.3010979	-0.8239645
C	-0.0531469	1.0479938	0.3489530
H	1.6660685	9.2113567	1.8840302
H	0.0947795	8.2612853	0.2566753
H	2.4496623	5.2811995	3.4271215
H	0.2131162	2.8546373	-3.0228562
H	-0.1643234	0.5727886	1.3150540
C	-0.4253919	5.5679075	-0.2110369
H	-0.8493481	6.3246529	-0.8705902
H	-1.2692739	5.0063543	0.2002082
O	3.1040306	7.7095069	3.6390832
C	3.3534692	9.0928038	3.7289544
H	4.0990851	9.2172372	4.5095784
H	3.7446132	9.4875180	2.7873006
H	2.4475795	9.6415438	4.0009548
C	-0.0172281	0.9777680	-2.0401502
C	2.4232220	-5.1695126	0.9080849
C	3.1714259	-5.4707343	-0.2193676
C	2.6552021	-5.3103711	-1.4958937

C	1.3632179	-4.8074844	-1.6473329
C	0.6170237	-4.4589826	-0.5251350
C	1.1312007	-4.6682404	0.7515975
H	2.8296724	-5.3219104	1.8997767
H	4.1752428	-5.8571539	-0.0996717
H	3.2408962	-5.5730393	-2.3676682
C	-0.7162267	-3.7662612	-0.6934121
C	-1.3729216	-4.2227615	-1.9759393
C	-1.6112999	-4.0850220	0.4822614
C	-0.5867566	-4.5777178	-3.0686879
C	-1.1868169	-4.8599145	-4.2958372
C	-2.5676338	-4.7971328	-4.4021645
C	-3.3651688	-4.4876107	-3.3113904
C	-2.7607848	-4.2079487	-2.0858075
H	-0.5771265	-5.1283003	-5.1492822
H	-3.0349356	-5.0137972	-5.3540457
H	-4.4437291	-4.4654312	-3.4020525
C	-2.9941002	-4.0714820	0.3226532
C	-3.8245367	-4.2152921	1.4337832
C	-3.2534891	-4.3874558	2.6851476
C	-1.8786421	-4.4466038	2.8526059
C	-1.0521531	-4.3019687	1.7385257
H	-4.9002516	-4.1930909	1.3138813
N	0.3328030	-4.3642201	1.8486167
H	0.6983818	-4.6185056	2.7515772
N	0.7929774	-4.6382656	-2.9048351
H	1.3189669	-4.9971936	-3.6847541
N	-3.5146759	-3.9085644	-0.9567422
H	-4.5128435	-4.0034988	-1.0481155
H	-3.8962065	-4.4955385	3.5492407
H	-1.4462945	-4.6047040	3.8324077
C	-0.3249636	-1.1136338	-0.7831670
C	-0.4987671	-2.3029686	-0.7490388
H	-0.0872305	0.4193083	-2.9640899

References

[1] Wahhab, A.; Therrien, E.; inventor; Methylgene Inc., assignee. Small Molecule Inhibitors of Protein Arginine methyltransferases. WO 2008104077 (A1). 2007-02-28.

[2] Laursen, B. W.; Krebs, F. C. *Chem. Eur. J.* **2001**, *7*, 1773-1783.

[3] Park, K.; Lee, B. M. inventor; Yuhan Corp., assignee. Novel phenylethynyl benzamide glucokinase activator and method for preparing same. WO 2014112798 (A1). 2013-01-16.

[4] Zhao, Y.; Truhlar, D. G. *Theor. Chem. Account* **2008**, *120*, 215-241. https://doi.org/10.1007/s00214-007-0310-x.

[5] Grimme, S.; Antony, J.; Ehrlich, S.; Krieg, H. *J. Chem. Phys.* **2010**, *132*, 154104. DOI: 10.1063/1.3382344

[6] Weigend, F.; Häser, M.; Patzelt, H.; Ahlrichs, R. *Chem. Phys. Lett.* **1998**, *294*, 143-152.

[7] Goerigk, L.; Hansen, A.; Bauer, C.; Ehrlich, S.; Najibi, A.; Grimme, S. *Phys. Chem. Chem. Phys.* **2017**, *19*, 32184–32215.

[8] Turbomole7.2: TURBOMOLE V7.2 2017, a development of University of Karlsruhe and Forschungszentrum Karlsruhe GmbH, 1989-2007, TURBOMOLE GmbH, since 2007; available from http://www.turbomole.com.
